# Predicting adverse drug event using machine learning based on electronic health records: a systematic review and meta-analysis

**DOI:** 10.3389/fphar.2024.1497397

**Published:** 2024-11-13

**Authors:** Qiaozhi Hu, Yuxian Chen, Dan Zou, Zhiyao He, Ting Xu

**Affiliations:** ^1^ Department of Pharmacy, West China Hospital, Sichuan University, Chengdu, Sichuan, China; ^2^ West China School of Medicine, Sichuan University, Chengdu, Sichuan, China; ^3^ Key Laboratory of Drug-Targeting and Drug Delivery System of the Education Ministry, Sichuan Engineering Laboratory for Plant-Sourced Drug and Sichuan Research Center for Drug Precision Industrial Technology, West China School of Pharmacy, Sichuan University, Chengdu, Sichuan, China

**Keywords:** systematic review, adverse drug event, prediction model, machine learning algorithm, electronic medical record data

## Abstract

**Introduction:**

Adverse drug events (ADEs) pose a significant challenge in current clinical practice. Machine learning (ML) has been increasingly used to predict specific ADEs using electronic health record (EHR) data. This systematic review provides a comprehensive overview of the application of ML in predicting specific ADEs based on EHR data.

**Methods:**

A systematic search of PubMed, Web of Science, Embase, and IEEE Xplore was conducted to identify relevant articles published from the inception to 20 May 2024. Studies that developed ML models for predicting specific ADEs or ADEs associated with particular drugs were included using EHR data.

**Results:**

A total of 59 studies met the inclusion criteria, covering 15 drugs and 15 ADEs. In total, 38 machine learning algorithms were reported, with random forest (RF) being the most frequently used, followed by support vector machine (SVM), eXtreme gradient boosting (XGBoost), decision tree (DT), and light gradient boosting machine (LightGBM). The performance of the ML models was generally strong, with an average area under the curve (AUC) of 76.68% ± 10.73, accuracy of 76.00% ± 11.26, precision of 60.13% ± 24.81, sensitivity of 62.35% ± 20.19, specificity of 75.13% ± 16.60, and an F1 score of 52.60% ± 21.10. The combined sensitivity, specificity, diagnostic odds ratio (DOR), and AUC from the summary receiver operating characteristic (SROC) curve using a random effects model were 0.65 (95% CI: 0.65–0.66), 0.89 (95% CI: 0.89–0.90), 12.11 (95% CI: 8.17–17.95), and 0.8069, respectively. The risk factors associated with different drugs and ADEs varied.

**Discussion:**

Future research should focus on improving standardization, conducting multicenter studies that incorporate diverse data types, and evaluating the impact of artificial intelligence predictive models in real-world clinical settings.

**Systematic Review Registration:**

https://www.crd.york.ac.uk/prospero/display_record.php?ID=CRD42024565842, identifier CRD42024565842.

## Introduction

Medical treatment can pose potential risks associated with drug-induced impairments. Adverse drug events (ADEs) are defined as injuries resulting from medical interventions related to drugs and may manifest as clinical signs, symptoms, or laboratory abnormalities (Bates et al., 1995). ADEs include not only adverse drug reactions but also medication errors, therapeutic failures, adverse medication withdrawal events, and overdoses (Nebeker et al., 2004). ADEs have been recognized as a significant challenge in contemporary clinical practice. ADEs are estimated to contribute to up to 4.5 million ambulatory encounters, 1.3 million emergency department visits, 350,000 hospitalizations, and 106,000 deaths annually in the United States (Sarkar et al., 2011; Food and Drug Administration FDA, 2024). Although many of these events occur unintentionally, some are preventable (Hartigan-Go and Wong, 2000). Therefore, predicting ADEs has become an area of active research.

Various methods have been used to predict ADEs, such as drug-drug interactions (Dmitriev et al., 2019), the chemical structures of drugs (Mantripragada et al., 2021), spontaneous reporting systems (Bae et al., 2020), and health records (Lippenszky et al., 2024). The prediction of potential adverse events through drug-drug interactions and chemical structures is based on pharmacological mechanisms but does not account for individual patient physiological conditions or specific diseases. Although spontaneous reporting systems can predict ADEs using patient information, this information is often incomplete. Compared to these approaches, using health records for ADE prediction is more clinically practical as they encompass comprehensive data across a patient’s hospitalization period. Moreover, with the advent of electronic health record (EHR) systems, these data can be rapidly compiled and analyzed to achieve more accurate ADE prediction.

Specific ADEs are often associated with particular medications and risk factors, and under certain conditions, specific medications tend to induce distinct categories of ADEs. For instance, antihypertensive medications generally do not cause nausea and vomiting, while chemotherapy in cancer patients often results in these side effects (Adel, 2017). Additionally, opioid-induced injuries are more likely to occur in geriatric patients over the age of 60 (Sacerdote, 2008; Khanna et al., 2020; Horrigan et al., 2023). Consequently, multiple studies have focused on predicting specific ADEs and their associations with drugs. Traditional statistical methods have encountered limitations due to the large number of risk factors (Ji et al., 2018), leading to the introduction of novel statistical approaches.

Machine learning (ML) is an interdisciplinary field within the broader domain of artificial intelligence that integrates statistics and computer science. ML is adept at managing complex non-linear relationships between variables and outcomes, offering high generalization capabilities and precision (Deo, 2015). It has been widely used to predict specific ADEs based on EHR data. However, systematic evaluations of these applications remain limited. Therefore, this systematic review aims to provide a comprehensive overview of the application of ML in predicting specific ADEs using EHR data.

## Method

This systematic review was conducted following the Preferred Reporting Items for Systematic Reviews and Meta-Analyses (PRISMA) guidelines (Moher et al., 2009). The review protocol was registered with PROSPERO (CRD42024565842). As this study used publicly available data, ethical approval was not required.

### Literature search and eligibility criteria

A systematic search was conducted across PubMed, Web of Science, Embase, and IEEE Xplore (containing a wealth of articles on biomedical engineering, medical devices, and health informatics) to identify relevant publications from their inception up to 20 May 2024. Search syntaxes were based on “machine learn” or “artificial intelligence” with “predict” and “adverse drug event or reaction”. Synonyms were determined based on MeSH terms and Emtree. The detailed study protocol on the search strategy and inclusion criteria were provided in the Supplementary Table S1.

### Study selection

Studies were included if they met the following criteria: 1) predicting specific ADEs or ADEs related to specific drugs, 2) applying ML algorithms based on EHRs, 3) providing sufficient explanations for research findings, and 4) publishing in English. The exclusion criteria were as follows: 1) studies focused on medical safety events rather than ADEs, 2) studies aimed at identifying and warning ADEs rather than predicting them, 3) studies that predict all ADEs in specific populations without sufficient specificity, 4) studies lacked a full-text version, 5) studies published in languages other than English, 6) studies that employed conventional algorithms, and 7) studies not based on EHR data.

### Screening process and data extraction

After removing duplicate studies, two independent reviewers (QZ Hu and YX Chen) evaluated the titles and abstracts to exclude studies that did not meet the eligibility criteria. The full texts of the potentially eligible studies were assessed. Any disagreements were resolved by consensus. The data extracted included the author, year, database, demographic characteristics, number of patients with ADEs, used ML algorithms, evaluation and validation performance metrics [e.g., accuracy, sensitivity, specificity, precision, F1 score, area under the curve of the receiver operating characteristic curve (AUC), and area under the precision-recall Curve (AUCPRC)], and risk factors before and after screening.

### Quality evaluation

Two reviewers independently assessed the quality of the included studies. Given that these retrospective studies employed medical artificial intelligence, we utilized two assessment tools to thoroughly evaluate both the appropriateness of the retrospective study design and the rigor of the artificial intelligence methodologies applied. The Agency for Healthcare Research and Quality (AHRQ) (AHQR, 2024) tool was used to evaluate the methodological quality and risk of bias in primary cross-sectional studies, while the Checklist for the Assessment of Medical AI (ChAMAI) (Cabitza and Campagner, 2021) was applied to assess the quality of artificial intelligence studies in the medical field. AHRQ tool consists of 11 items, each rated as “yes” or “no”, with corresponding scores of 1 or 0, respectively (AHQR, 2024). The maximum AHRQ score is 11 points. ChAMAI includes 30 items in six dimensions: problem understanding, data understanding, data preparation, modeling, validation, and deployment (Cabitza and Campagner, 2021). Each question is classified as high-priority (scored 0, 1, or 2) or low-priority (scored 0, 0.5, or 1), with a maximum possible score of 50 points (Cabitza and Campagner, 2021).

### Statistical analysis

The effects and 95% confidence intervals (CIs) were estimated using random effects models. Pooled sensitivity, specificity, diagnostic odds ratios (DORs), and their respective 95% CIs were calculated based on contingency tables. Overall performance was assessed using the summary receiver operating characteristic (SROC) curve and the area under the SROC curve (AUC). Publication bias was evaluated using the Deek funnel plot test. Pooled sensitivity, specificity, and DORs were calculated using Meta-DiSc 2.0, while publication bias was analyzed with Stata 16.0 software.

## Results

### Characteristics of the included studies

**FIGURE 1:**
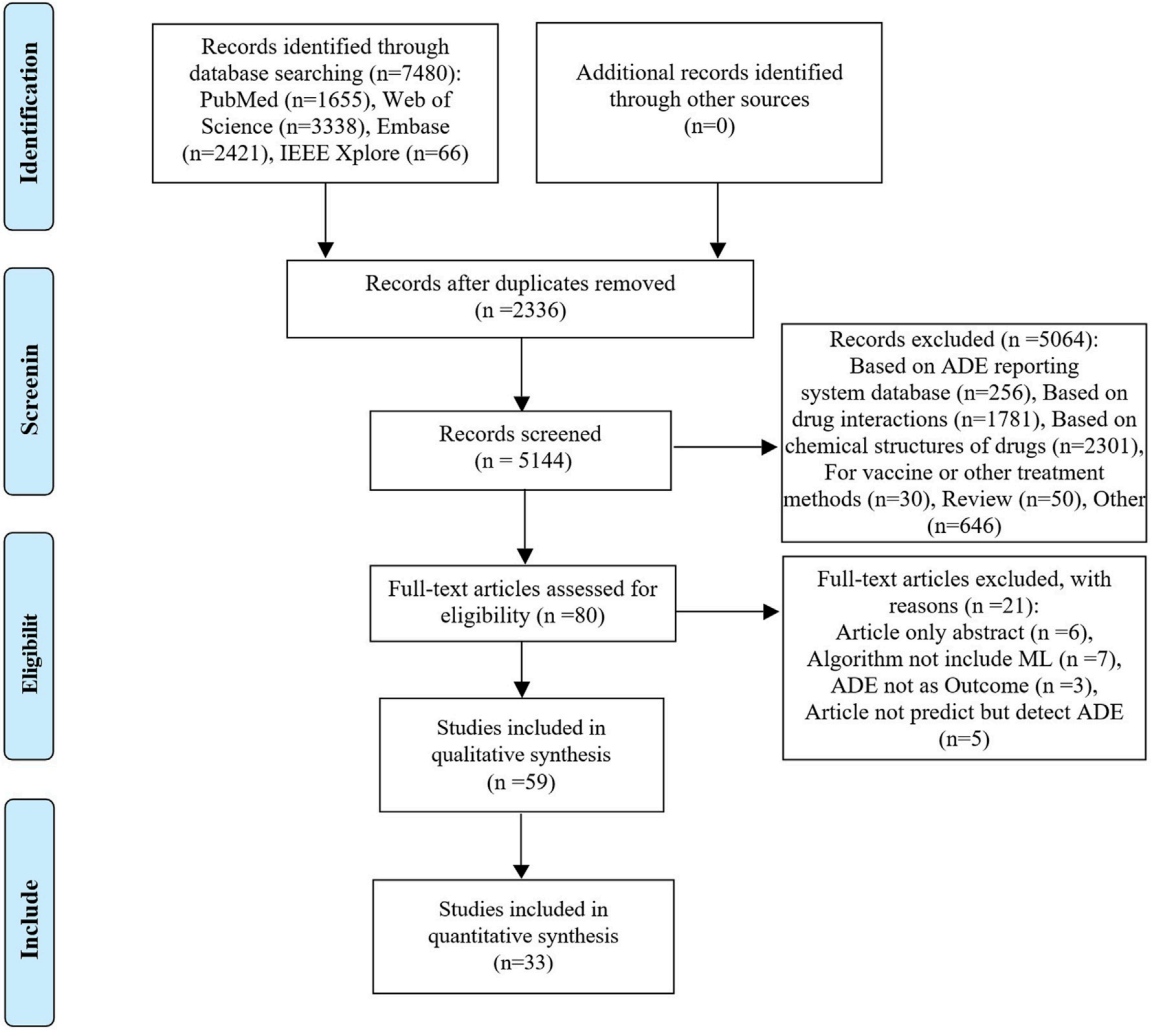
PRISMA flow diagram of the citation search and selection strategy. ADE: adverse drug event; PRISMA: Preferred Reporting Items for Systematic reviews and Meta-Analyses.

The database search yielded 7,480 relevant studies. After removing duplicates, the titles and abstracts were screened for relevance. Subsequently, the full texts of 80 studies were reviewed, with 59 studies (Liu et al., 2018; Dong et al., 2019; Imai et al., 2019; Imai et al., 2020; Lai et al., 2020; Sun et al., 2020; Yoo et al., 2020; Heilbroner et al., 2021; Herrin et al., 2021; Kim W. et al., 2021; Kim J. S. et al., 2021; Lewinson et al., 2021; Satheeshkumar et al., 2021; Simon et al., 2021; Zhou et al., 2021; 2023; Chang et al., 2022; Huang et al., 2022; Huang et al., 2023; Li et al., 2022; Mu et al., 2022; Okawa et al., 2022; On et al., 2022; Venäläinen et al., 2022; Wu et al., 2022; Xu et al., 2022; Asai et al., 2023; Chambers et al., 2023; Chen et al., 2023; Cheng et al., 2023; Gong et al., 2023; Goyal et al., 2023; Güven et al., 2023; Heo et al., 2023; Hu et al., 2023; Jeong et al., 2023; Jiang et al., 2023; Lee et al., 2023; Liao et al., 2023; Lu et al., 2023; Ma J. et al., 2023; Ma X. et al., 2023; Mao et al., 2023; Maray et al., 2023; Mora et al., 2023; Ruiz Sarrias et al., 2023; Yan et al., 2023; Zhang et al., 2023; Chiu et al., 2024; Choi et al., 2024; Lippenszky et al., 2024; Matsumoto et al., 2024; Nguyen et al., 2024; Noda et al., 2024; Patel et al., 2024; Surendran et al., 2024; Xiao et al., 2024; Yagi et al., 2024; Zhao et al., 2024) included in the qualitative synthesis. Among the 59 studies included, 33 studies that provided extractable contingency tables could been included in the quantitative synthesis, as illustrated in Figure 1.

**FIGURE 2:**
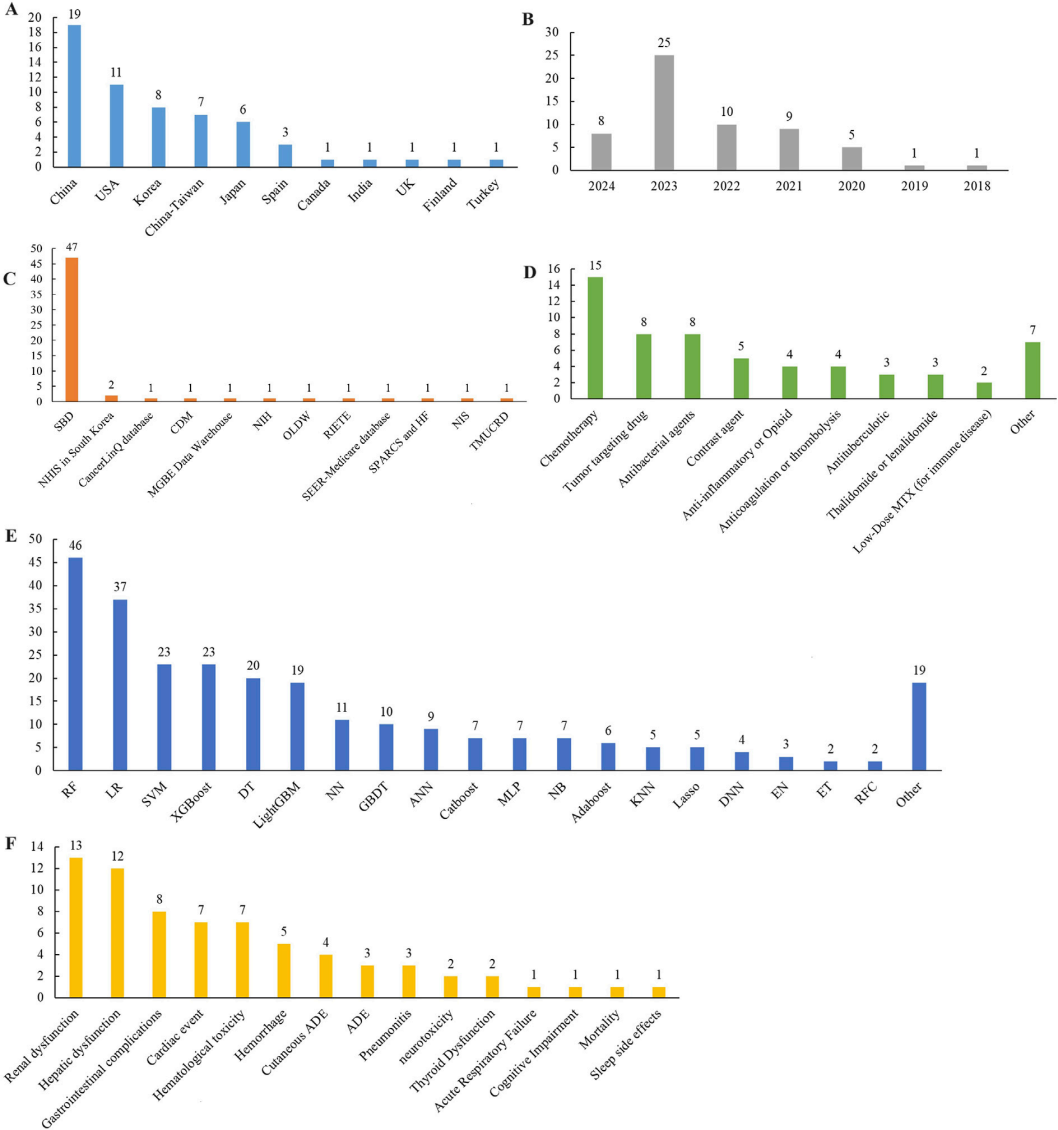
Distribution of included studies in **(A)** region, **(B)** publication year, **(C)** database, **(D)** medicine, **(E)** ML models, and **(F)** ADE. Location: United States, the United States, United Kingdom, the United Kingdom. Database: SBD, Self-built database; NIS, National Inpatient Sample; TMUCRD, Taipei Medical University Clinical Research Database; MGBE Data Warehouse, Massachusetts General Brigham Enterprise Data Warehouse; NHIS, National Health Insurance Service; NIH, National Institutes of Health; SPARCS, New York State Statewide Planning and Research Cooperative System; HF, Health Facts; RIETE, Registro Informatizado de Enfermedad TromboEmbólica; OLDW, OptumLabs Data Warehouse; CDM, Common Data Model. Drugs that cause ADE: MTX, methotrexate; ADE, Adverse drug event. ML Algorithms: RF, random forest; LR, Logistic regression; Lasso, Least Absolute Shrinkage and Selection Operator; EN, Elastic Net; XGBoost, eXtreme Gradient Boosting; DT, decision tree; GBDT, gradient boosting decision tree; LightGBM, light gradient boosting machine; AdaBoost, Adaptive Boosting, CatBoost, categorical boosting; NN, Neural network; ANN, Artificial Neural Network; SVM, Support vector machine; KNN, K-Nearest Neighbors; NB, Naïve Bayes; ET, extremely random tree; DNN, Deep neural networks; MLP, Multi-Layer perceptron; RFC, Random forest classification.

Most included studies were conducted in China (n = 19), followed by contributions from the United States, Korea, Taiwan, and Japan. These studies were published between 2018 and 2024, with most published in 2023. Ten studies were based on government or commercial databases from the United States, Korea, and Spain, while 49 used self-constructed databases based on hospital patient data. A total of 15 drug categories were identified, with most studies focusing on chemotherapy (n = 15), followed by tumor-targeting drugs, antibacterial agents, and contrast agents. ADEs in the included studies were classified into 15 categories, with the most predicted being renal dysfunction (n = 13), followed by hepatic dysfunction, gastrointestinal complications, cardiac events, and hematologic toxicity. 38 mL algorithms were reported, with random forest (RF) being the most frequently used, followed by support vector machine (SVM), eXtreme gradient boosting (XGBoost), decision tree (DT), and light gradient boosting machine (LightGBM). Additionally, logistic regression (LR), a traditional statistical method, was reported 37 times. Detailed information is provided in Table 1 and Figure 2.

**TABLE 1:** Characteristics of included studies.

Classification of drugs	Study	Location	No. Of patients	Database	Age	No. Of patient with ADE (%)	Drugs that cause ADE	Type of ADE	No of initial features	ML algorithms	Evaluation and validation	Best performance
Tumor targeting drug	Lippenszky et al. (2024)	United States	2,230	SBD	63.00 (54.00–70.00)	Pneumonitis:190 (9.14%), Hepatitis:329 (16.84%), Colitis:160 (7.49%)	ICI	Pneumonitis, Hepatitis, Colitis	234	RF	①	Pneumonitis-AUC:0.739, Hepatitis -AUC:0.729 Colitis-AUC:0.755
	Cheng et al. (2023)	China	141	SBD	Age ≥ 65: n = 45; Age < 65:n = 96	40 (28.37%)	ICI	Pneumonitis	20	CNN + LR	①	AUC: 0.9
	Gong et al. (2023)	China	190	SBD	non-ADE: 58.00 (52.00–67.00); ADE: 61.00 (54.75–67.00)	48 (25.26%)	ICI	Pneumonitis	55	EN	①②	AUC: 0.81
	Heilbroner et al. (2021)	United States	3,229	CancerLinQ database	64.40 ± 10.80	301 (9.32%)	ICI	Cardiac event	356	XGBoost	①	AUC: 0.65
	Jiang et al. (2023)	China	121	SBD	65.00 (56.00–73.00)	47 (38.84%)	BTK inhibitors	Hematological toxicity	36	DT, RF, GBDT, XGBoost, LightGBM	①	XGBoost-AUC:0.671
	Kim et al. (2021b)	Korea	187	SBD	Age ≥ 65: n = 112; Age < 65: n = 75	23 (12.30%)	ICI	Thyroid-Related ADE	10	LR, EN, RF, SVM (linear or radial)	①②	RF-AUC: 0.77
	Lewinson et al. (2021)	Canada	142	SBD	non-ADE: 64.80 ± 11.30, ADE:68.10 ± 11.50	54 (13.17%)	ICI	Cutaneous ADE	10	RF	①	AUC: 0.765
	Kim et al. (2021a)	Korea	353	SBD	Age ≥ 60: n = 169; Age < 60:n = 184	144 (40.79%)	Nilotinib	Hepatic dysfunction	17	MLR, EN, RF, SVM (linear or radial)	①②	MLR- AUC:0.65 EN-AUC:0.65
Low-Dose MTX (for immune disease)	Surendran et al. (2024)	India	569	SBD	non-ADE: 52.33 ± 13.27, ADE: 53.19 ± 10.61	104 (18.28%)	MTX	Hepatic dysfunction	22	RF	①②	RF (full-feature)-AUC:0.658
	Hu et al. (2023)	China	782	SBD	47.85 ± 15.56	279 (35.68%)	MTX	Hepatic dysfunction	17	XGBoost, AdaBoost, CatBoost, GBDT, LightGBM, TPOT, RF, ANN	①	RF-AUC:0.97
Chemotherapy	Chambers et al. (2023)	United Kingdom	1,214	SBD	hospital 1:55.00 (18.00–88.00); hospital 2: 60.00 (18.00–88.00)	Renal: 38 (3.12%); Hepatic146 (12.03%)	Chemotherapy	Renal and Hepatic dysfunction	N	MLP	①②	MLP (Renal dysfunction)-AUC:0.76 MLP (Hepatic dysfunction)-AUC:0.72
	Chang et al. (2022)	China-Taiwan	211	SBD	55.80 ± 10.28	31 (14.69%)	Anthracycline Chemotherapy	Cardiac event	15	RF, SVM, KNN, LightGBM, MLP	①	MLP-AUC:0.664
	Huang et al. (2022)	China-Taiwan	118	SBD	non-ADE: 66.69 ± 11.17 ADE:61.61 ± 11.39	28 (23.73%)	Platinum	Renal dysfunction	84	ANN, LR, RF, SVM	①②	ANN (integrated)-AUC:0.900
	On et al. (2022)	Korea	935	SBD	60.90 ± 12.10	NV: 448 (47.9%), FA:441 (47.2%) DI: 171 (18.3%) PN: 203 (21.7%) HS:85 (9.1%) ST: 91 (9.7%) HFS:71 (7.6%) CO:71 (7.6%)	Chemotherapy	NV, FA, DI, PN, HS, ST, HFS, CO	35	LR, DT, NN	②	DT (NV) - AUC: 0.81 DT (FA) - AUC: 0.72 LR (DI)-AUC: 0.71 DT (PN) - AUC: 0.67 LR (HS)-AUC: 0.76 DT (ST) - AUC: 0.83 LR (HFS)-AUC: 0.74 DT (CO) - AUC: 0.88
	Ruiz Sarrias et al. (2023)	Spain	267	SBD	63.71 ± 10.76	Leukopenia 8/267 Lymphopenia 13/267 Mucositis 3 (1.12%) Neutropenia 66 (24.72%) Thrombocytopenia 5 (1.87%)	5FU-Based Chemotherapy	Hematological toxicity	39	BN	①②	AUC: NA Accuracy: 0.8 Sensitivity: 0.71
	Satheeshkumar et al. (2021)	United States	21,879	NIS	non-ADE:53.90 ± 17.90 ADE: 50.70 ± 17.20	253 (1.2%)	Chemotherapy	Ulcerative mucositis	N	Lasso, GBM	①②	GBM-AUC:0.79
	Venäläinen et al. (2022)	Finland	5,879	SBD	Age 0–40: n = 280 Age 40–65: n = 2,864 Age 65-: n = 2,727	262 (4.46%)	Chemotherapy	Neutropenic infection	55	Lasso	①	AUC: 0.84
	Zhang et al. (2023)	China	756	SBD	64.00 (19.00–85.00)	vomiting: 17 (2.3%) nausea: 57 (7.6%)	Chemotherapy	NV	19	RF, SVM, Catboost, NN, DT, DF	①②	Deep forest-AUC:0.850
	Zhou et al. (2021)	China	386	SBD	non-ADE: 63.30 ± 9.59 ADE: 68.03 ± 10.44	117 (30.31%)	Chemotherapy	Cognitive Impairment	14	LASSO, RF, SVM	①②	LASSO-AUC: 0:799
	Li et al. (2022)	United States	36,030	SEER-Medicare database	Training dataset: 71.56 ± 9.19 Testing dataset:74.96 ±8.27	6,753 (18.74%)	FU-Based Chemotherapy	Cardiac event	30	XGBoost, RF, LR	①②	XGBoost-AUC:0.816
	Matsumoto et al. (2024)	Japan	186	SBD	non-ADE:71.00 (38.00–90.00) ADE:73.00 (48.00–83.00)	46 (24.73%)	Gemcitabine Plus Platinum	Thrombocytopenia	29	EM combine with GBDT	②	AUC: 0.76
	Huang et al. (2023)	China	3,315	SBD	57.00 (49.00–63.00)	121 (3.65%)	Oxaliplatin	Hepatic dysfunction	22	ANN, LR	①	ANN-AUC: 0.920
	Nguyen et al. (2024)	China-Taiwan	1,321	TMUCRD	56.00 ± 11.30	112 (8.5)	Chemotherapy	Cardiac event	5 categories	ANN, LR	①②	ANN-AUC: 0.897
	Yagi et al. (2024)	United States	1,011	MGBE Data Warehouse	57.10 ± 16.40	88 (8.70%)	Anthracycline Chemotherapy	Cardiac event	N	AI-EF model	①	AUC:0.78
	Okawa et al. (2022)	Japan	1,240	SBD	Training dataset: 62.50 ± 11.00 Testing dataset: 63.80 ± 11.60	213 (17.18%)	Platinum	AKI	8	NN combine with GBDT	①	AUC:0.67
Antibacterial agents	Imai et al. (2019)	Japan	396	SBD	non-ADE: 60.00 (18.00–91.00) ADE:56.00 (19.00–96.00)	61 (15.40%)	Ganciclovir	Neutropenia	35	LR, DT	①②	LR-Accuracy:0.846 DT-Accuracy:0.846
	Mu et al. (2022)	China	724	SBD	51.00 (39.00–63.00)	86 (11.88%)	Vancomycin	AKI	51	XGBoost	①	AUC:0.879
	Imai et al. (2020)	Japan	1,141	SBD	65.00 (18.00–96.00)	179 (15.7%)	Vancomycin	Renal dysfunction	8	ANN	②	ANN-AUC:0.83
	Zhao et al. (2024)	China	564	SBD	non-ADE:48.32 ± 20.00 ADE:60.64 ± 22.31	89 (15.78%)	Linezolid	Thrombocytopenia	20	ANN, LR	①	LR-AUC:0.9796
	Patel et al. (2024)	United States	2,217	SBD	53.00 ± 18.60	10,346 (7.67%)	Linezolid	Thrombocytopenia and anemia	53	RFC	①	RFC(Thrombocytopenia)-AUC:0.905 RFC(Anemia)-AUC: 0.783
	Asai et al. (2023)	Japan	630	SBD	78.00 (67.00–85.00)	127 (20.16%)	Carbapenem	Hepatic dysfunction	24	DT	①②	AUC: NA Accuracy: 0.825
	Ma et al. (2023a)	China	318	SBD	61.00 (49.00–69.00)	70 (22.01%)	Voriconazole	Hepatic dysfunction	N	AdaBoost, DT, GBDT, LR, NN, RF	①②	LR-AUC:0.7933
	Chiu et al. (2024)	China-Taiwan	1,391	SBD	Training dataset: non-ADE-74.06 ± 15.70, ADE-75.00 ± 15.55 Testing dataset: non-ADE-73.89 ± 14.24, ADE-69.91 ± 15.61	524 (37.64%)	Colistin	Renal dysfunction	55	Catboost, LightGBM, RF with SMOTE, SVM-SMOTE, Tomek or SMOTETL	①②	Catboost (SVM-SMOTE)-AUC:0.786
Antituberculotic	Lai et al. (2020)	China-Taiwan	127	SBD	non-ADE:55.50 ± 21.40 ADE: 64.00 ± 17.90	21 (16.53%)	Antituberculotic	Hepatic dysfunction	19	ANN, SVM, and RF with traditional parameters, genetic parameters or combined parameters	①②	ANN (with combined parameters)-AUC:0.898
	Xiao et al. (2024)	China	7,071	SBD	none-ADE:47.00 ± 19.00 ADE: 48.00 ± 18.00	1,151 (16.28%)	Antituberculotic	Hepatic dysfunction	424	LR, RF, XGBoost	①②	XGBoost-AUC:0.887
	Liao et al. (2023)	China-Taiwan	2,248	SBD	67.70 ± 16.40	Hepatic dysfunction: 871 (38.7%) ARF:107 (4.8%) Mortality: 120 (5.3%)	Antituberculotic	Hepatic dysfunction ARF Mortality	36	MLP, RF, LightGBM, XGBoost, SVM, LR	①②	XGBoost-AUC (Hepatic dysfunction): 0.92, RF-AUC(ARF): 0.884, MLP-AUC (Mortality): 0.834
Anti-inflammatory or Opioid	Jeong et al. (2023)	Korea	30,808	NHIS in South Korea	non-ADE: 55.44 ± 14.91, ADE: 58.91 ± 13.59	1,229 (3.99%)	Anti-inflammatory	Gastric Ulcer	38	LR, SVM, RF, GBM, XGBoost	①②	LR-AUC:0.636 SVM-AUC:0.637 RF-AUC:0.862 GBM-AUC:0.896 XGBoost-AUC:0.893
	Lee et al. (2023)	Korea	344,487	NHIS in South Korea	Derivation cohort (non-ADE: 75.65 ± 6.37, ADE: 78.47 ± 6.58) External validation cohort (non-ADE: 71.83 ± 5.66, ADE: 75.38 ± 5.11)	8,548 (2.48%)	Anti-inflammatory	Gastrointestinal complications	17	LASSO	①②	AUC:0.79
	Liu et al. (2018)	United States	4,350	NIH	45.00–79.00	371 (8.53%)	Analgesics	Cardiac event	300	LR, SVM, DT, GBDT, XGBoost	②	XGBoost-AUC:0.92
	Dong et al. (2019)	United States	SPARCS: 440,000 HF: 110,000	SPARCS and HF	N	50,000 (9.09%)	Opioid	ADE	SPARCS: 4,004; HF: 8,403	RF, LR, DT, DNN	N	RF (SPARCS)-AUC: 0.9494 DL (Health Facts)-AUC: 0.9541
Anticoagulation or thrombolysis	Xu et al. (2022)	China	345	SBD	non-ADE:70.00 (63.00–80.00) ADE: 78 .00 (68.00–82.00)	45 (13.04%)	Intravenous thrombolysis	Hemorrhage	64	LR, RF	①	LR-AUC: 0.795
	Mora et al. (2023)	Spain	Learning: 49,587; Validation: 10,337	RIETE	Training dataset: ADE-75.00 (65.00–83.00), no-ADE-68.00 (53.00–79.00) Testing dataset: ADE-70.00 (59.00–80.00), no-ADE-66.00 (53.00–77.00)	Learning:837 (1.76%) Validation:227 (2.19%)	anticoagulation	Hemorrhage	55	XGBoost	①②	AUC:0.91
	Chen et al. (2023)	China	798	SBD	Age < 80: n = 116 Age ≥ 80: n = 682	112 (14.0%)	Rivaroxaban	Hemorrhage	26	XGBoost	①②	XGBoost-AUC: 0.776
	Herrin et al. (2021)	United States of America	306,463	OLDW	69.00 ± 12.60	12,322 (4.02%)	Antithrombotic Treatment	Gastrointestinal hemorrhage	32	RegCox, RSF, XGBoost	①②	XGBoost-AUC:0.66 RSF-AUC:0.60 RegCox-AUC:0.66
Contrast agent	Choi et al. (2024)	Korea	38,481	SBD	non-ADE: 62.40 ± 11.20 ADE: 66.40 ± 12.40	1,185 (3.1%)	Contrast agent	AKI	N	Lasso, GBM, RF, DT, Adaboost	①②	GBM-AUC:0.875
	Zhou et al. (2023)	China	2,230	SBD	non-ADE: 70.00 (61.00–78.00) ADE:75.00 (67.25–81.00)	120 (5.38%)	Contrast agent	AKI	51	LR, RF, GBDT XGBoost, NB	①	GBDT-AUC:0.755
	Sun et al. (2020)	China	1,495	SBD	66.60 ± 13.90	226 (15.1%)	Contrast agent	AKI	N	RF, LR, SVM	①②	RF-Accuracy:0.721
	Yan et al. (2023)	China	4,218	SBD	Training dataset: 64.80 ± 13.40 Testing dataset: 64.80 ± 13.20 External validation cohort: 66.90 ± 14.50	440 (10.43%)	Contrast agent	AKI	14	DNN, LR	①	DNN-AUC:0.94
	Ma et al. (2023b)	China	240	SBD	63.00 (54.00–68.00)	37 (15.41%)	Contrast agent	Renal dysfunction	45	LR, SVM, RF, XGBoost, DT	①②	SVM-AUC:0.784
Thalidomide or lenalidomide	Mao et al. (2023)	China	164	SBD	34.30 ± 12.70	59 (36%)	Thalidomide	Peripheral neuropathy	168	XGBoost, ET, GBDT, RF, LR	①②	LR-AUC: 0.907
	Maray et al. (2023)	Spain	64	SBD	76.00 (69.00–81.00)	44 (68.75%)	Lenalidomide-dexamethasone	ADE	23	LR, CART	①	LR-AUC: 0.91
	Yoo et al. (2020)	Korea	119	SBD	Training dataset: 9.50 ±2.60 Testing dataset: 8.50 ±2.50	25 (21.00%)	Methylphenidate	Sleep side effects	34	LR, SVM, J48	①②	LR-AUC: 0.92
Other	Lu et al. (2023)	China-Taiwan	6,497	SBD	Training dataset: non-ADE-73.00 (62.00–83.00), ADE-76.00 (66.00–83.00); Testing dataset: non-ADE-72.00 (61.00–81.00), ADE-75.00 (64.00–83.00)	858 (13.21%)	Amiodarone	Thyroid Dysfunction	46	XGBoost, AdaBoost, KNN, LR with or without borderline-SMOTE, ENN, and B-SMT-ENN	①②	XGBoost + ENN-AUC:0.939
	Simon et al. (2021)	United States	35,639	SBD	non-ADE: 54.34 ± 18.81 ADE: 58.78 ± 17.45	4,558 (12.79%)	QT-prolonging medication	Long QT syndrome	8	RF, LR, NB, DNN	①	DNN-AUC: 0.71
	Wu et al. (2022)	China	530	SBD	Age ≤ 44: n = 121 45 ≤ Age ≤ 59: n = 193 60 ≤ Age ≤74: n = 13, Age≥ 75: n = 84	106 (20.00%)	Chinese herbal injections	ADE	83	AdaBoost, Bagging, Bernoulli NB, DT, EM, ET, Gaussian NB, GBM, KNN, LDA, LR, Multinomial NB, Passive Aggressive, QDA, RF, SGD, SVM, XGBoost	①②	EM-AUC:0.793
	Goyal et al. (2023)	United States	Cl:2,159, Wa: 1855, Ci:3,151, Es:2,597, Fluo: 2,719, Fluv:117, Pa:1,100, Se:4,052, Vo:149, Combined SSRI: 10,362	SBD	Cl:63.10 ± 10.40 Wa: 60.20 ± 11.60 Ci:50.40 ± 14.30 Es:50.20 ± 15.50 Fluo: 48.10 ± 14.80 Fluv: 43.30 ± 14.20 Pa:51.60 ± 13.30 Se:49.00 ± 15.60 Vo:49.00 ± 15.60 Combined SSRI: 49.40 ± 15.00	Cl:234 (10.8%) Wa: 293 (15.8%) Ci:286 (9.1%) Es: 156 (6.0%) Fluo: 226 (8.3%) Fluv:23 (19.6%) Pa: 97 (8.8%) Se: 336 (8.3%) Vo: 30 (20.0%) Combined SSRI: 996 (9.6%)	SSRI	Hemorrhage	88	LR, DT, RF, XGBoost	①②	LR (Cl)-AUC:0.638 XGBoost (Wa)-AUC:0.682 RF-AUC(Ci):0.698 RF(Es)-AUC:0.656 DT (Fluo)-AUC:0.664 XGBoost (Fluv)-AUC:0.771 RF(Pa)-AUC:0.632 RF(Se)-AUC:0.665 LR (Vo)-AUC:0.802 XGBoost (Combined SSRI)-AUC:0.688
	Heo et al. (2023)	Korea	15,236	CDM	N	3,809 (25.00%)	ARB	Hepatic dysfunction	133	IMV-LSTM module	①	AUC: 0.87
	Güven et al. (2023)	Turkey	409	SBD	58.50 ± 10.60	50 (12.24%)	ACEI	Renal dysfunction	19	RF, KNN, NB, XGBoost, SVM, NN, LR	①②	XGBoost-AUC: 0.99
	Noda et al. (2024)	Japan	204	SBD	non-ADE:59.00 (18.00–88.00) ADE:58.00 (18.00–84.00)	33 (16.18%)	Tacrolimus	Renal dysfunction	23	1)LR with no regularization backward, 2) selection of features, 3) Lasso, 4) ridge regularization, 5) both ridge regularization and backward selection of features, 6) SVM (linear), 7) SVM (polynomial), 8) SVM (Gaussian), 9) Adaboost, 10) Logitboost, 11) random under sampling boost, 12) RF, 13) NN, with or without SMOTE	①②	Logitboost (SMOTE + Feature Selection)-AUC:0.801

Location: United States, the United States; United Kingdom, the United Kingdom. Database: SBD, Self-built database; NIS, national inpatient sample; TMUCRD, taipei medical university clinical research database; MGBE, data warehouse, Massachusetts General Brigham Enterprise Data Warehouse; NHIS, national health insurance service; NIH, national institutes of health; SPARCS, new york state statewide planning and research cooperative system; HF, health facts; RIETE, Registro Informatizado de Enfermedad TromboEmbólica, OLDW, OptumLabs Data Warehouse, CDM, Common Data Model. Drugs that cause ADE: ICI, immune checkpoint inhibitor; BTK, Bruton’s tyrosine kinase MTX, methotrexate, Cl, Clopidogre, Wa, Warfarin, Ci, Citalopram, Es, Escitalopram, Fluo, Fluoxetine, Fluv, Fluvoxamine, Pa, Paroxetine, Se, Sertraline, Vo, Vortioxetine, Type of ADE: SSRI, selective serotonin reuptake inhibitor; NV, nausea-vomiting; FA, fatigue-anorexia; DI, diarrhea; PN, peripheral neuropathy; HS, hypersensitivity; ST, stomatitis; HFS, hand-foot syndrome; CO, constipation; AKI, Acute Kidney Injury ADE, Adverse drug event; ML, Algorithms; RF, random forest; CNN, convolutional neural network; RFC, random forest classification; LR, logistic regression, Lasso, Least Absolute Shrinkage and Selection Operator; EN, elastic net, XGBoost, eXtreme Gradient Boosting; DT, decision tree; GBDT, gradient boosting decision tree, LightGBM, light gradient boosting machine, AdaBoost, Adaptive Boosting, CatBoost, categorical boosting, TPOT, Tree-based Pipeline Optimization Tool; NN, neural network; ANN, artificial neural network; SVM, support vector machine; MLR, multivariate logistic regression; MLP, Multi-Layer perceptron, KNN, K-Nearest Neighbors, BN, bayesian network; NB, Naïve Bayes; DF, deep forest; EM, ensemble model; AI-EF, model; AI, model detecting reduced left ventricular ejection fraction from 12-lead electrocardiograms (ECG), RegCox, Cox regression, RSF, random survival forests; ET, extremely random tree; CART, classification and regression trees; GBM, gradient boosting machine; DNN, deep neural networks; LDA, latent dirichlet allocation; QDA, quadratic discriminant analysis; SGD, stochastic gradient descent; STROBE, strengthening the reporting of observational studies in epidemiology; ENN, edited nearest neighbor, B-SMT-ENN, borderline synthetic minority oversampling technique–edited nearest neighbor; SMOTE, synthetic minority oversampling technique, Tomek, Tomek links, SMOTETL, SMOTE, and Tomek link, SVM-SMOTE, SVM, and SMOTE, IMV-LSTM, interpretability multivariate long short-term memory. Evaluation and validation: ①Training and Testing ②K-fold cross-validation. Performance metrics: AUC, area under the receiver operating characteristic curve statistic.

Among the 59 included studies, 6 (Sun et al., 2020; Satheeshkumar et al., 2021; Chambers et al., 2023; Ma J. et al., 2023; Choi et al., 2024; Yagi et al., 2024) did not provide any information on initial features, while 53 reported initial features associated with ADEs. Of these, 46 studies detailed the types and the number of initial features, while 7 studies (Dong et al., 2019; Heilbroner et al., 2021; Li et al., 2022; Güven et al., 2023; Zhou et al., 2023; Matsumoto et al., 2024; Xiao et al., 2024) reported only the number of initial features. The initial number of features used to build the models ranged from 8 to 8,403. The commonly identified features included demographic data, treatment information, laboratory results, and disease history. Additionally, genes, plasma concentration, and pharmacokinetics were considered risk factors in some studies (Lai et al., 2020; Huang et al., 2022; Ruiz Sarrias et al., 2023). Of the 59 included studies, 47 reported conducting feature selection to identify the most important predictors for their models, while 10 did not perform feature screening. Two studies (Chambers et al., 2023; Ma J. et al., 2023) did not provide details on initial or significant features. Further details are provided in Supplementary Table S2.

### Evaluating the quality of studies

The risk of bias assessment using the AHRQ tool indicated that the overall quality of the studies was generally high, with an average score of 9.254 points. Among the included studies, 30 scored above 10 points, 28 scored between 7 and 9 points, and one study (Lewinson et al., 2021) scored 4 points. Items 8 and 11 had the lowest average scores, 0.424 and 0.322 points, respectively, while the average scores for the other items were considerably higher. Detailed information is provided in Supplementary Table S3.

According to the ChAMAI checklist, the overall average score of the included studies was 32.48, with scores ranging from 22.00 to 40.00 (Supplementary Table S4; Supplementary Figure S1). Of the included studies, 39 scored higher than 30 points, 19 scored between 25 and 29 points, and one study (Lewinson et al., 2021) scored below 25. Regarding specific categories, the highest scoring rates were observed in modeling and problem understanding, at 100% and 99.5%, respectively, while data preparation had the lowest scoring rate, at 37.25%.

### Predictive performance for ML methods

AUC was a critical metric to assess model performance. Fifty-four studies reported AUC values, with an average AUC of 76.68% ± 10.73, indicating generally favorable performance in all models evaluated. Artificial neural networks (ANN), gradient boosting machines (GBM), CatBoost, and XGBoost demonstrated high AUC values, with average values exceeding 80%. The RF algorithm, though the most frequently reported, had a slightly lower average AUC of 77.00% ± 7.83. The impact of feature selection on model performance was examined in three studies (Sacerdote, 2008; Goyal et al., 2023; Noda et al., 2024), which showed that, in most cases, feature selection improved model performance.

**FIGURE 3:**
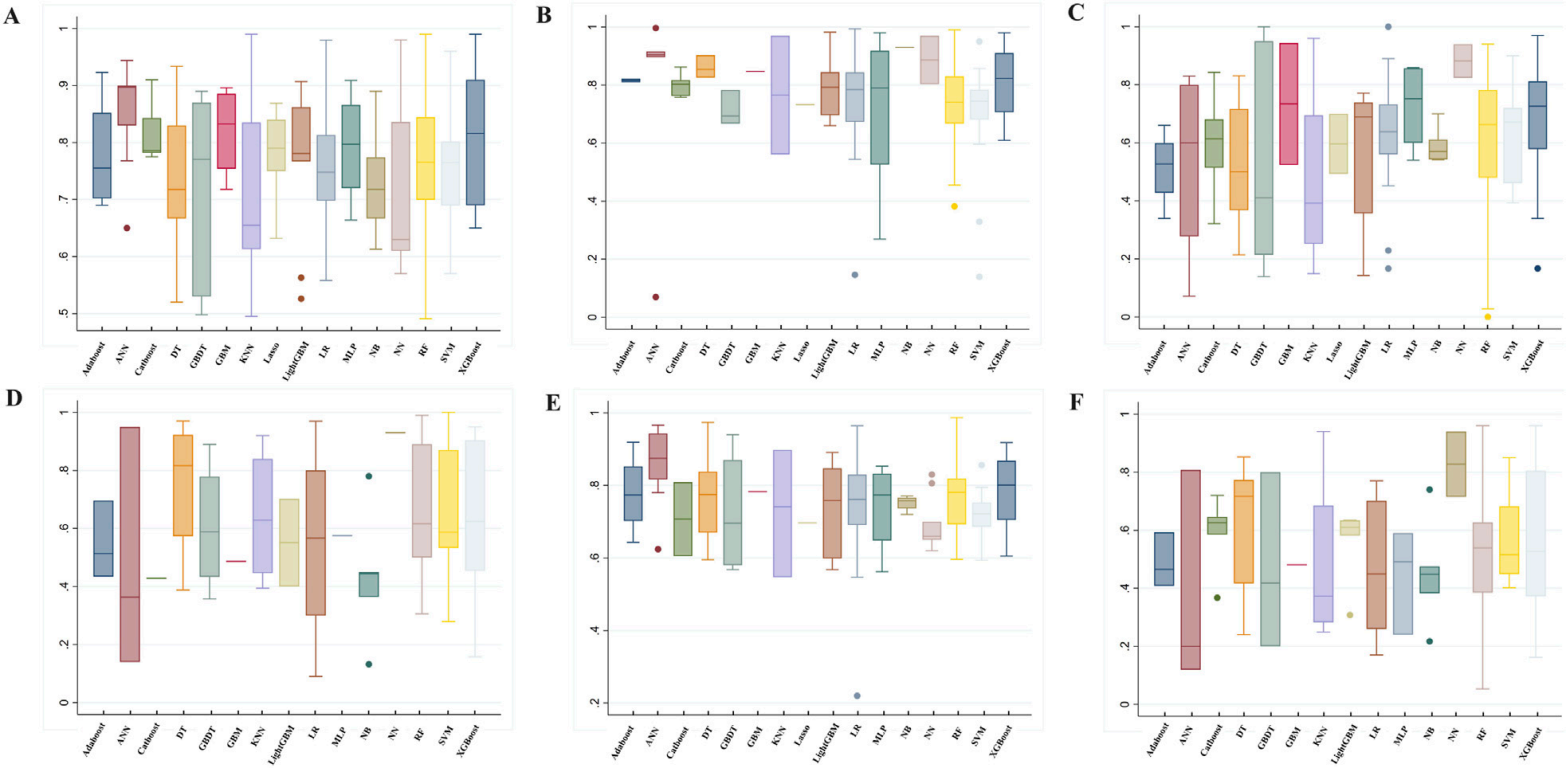
Summary of prediction model performance. **(A)** AUC, **(B)** Specificity, **(C)** Sensitivity (recall), **(D)** Precision, **(E)** Accuracy, **(F)** F1 ML Algorithms: RF, random forest; LR, Logistic regression; Lasso, Least Absolute Shrinkage and Selection Operator; XGBoost, eXtreme Gradient Boosting; DT, decision tree; GBDT, gradient boosting decision tree; LightGBM, light gradient boosting machine; AdaBoost, Adaptive Boosting; CatBoost, categorical boosting; NN, Neural network; ANN, Artificial Neural Network; SVM, Support vector machine; KNN, K-Nearest Neighbors; NB, Naïve Bayes; GBM, gradient boosting machine; MLP, Multi-Layer perceptron.

In addition to AUC, model performance was evaluated using several other metrics: accuracy (76.00% ± 11.26), precision (60.13% ± 24.81), sensitivity (62.35% ± 20.19), specificity (75.13% ± 16.60), and F1 score (52.60% ± 21.10). Detailed metrics for the machine learning algorithms are presented in Figure 3 and Supplementary Table S5.

### Meta-regression

#### Pooled analysis

**FIGURE 4:**
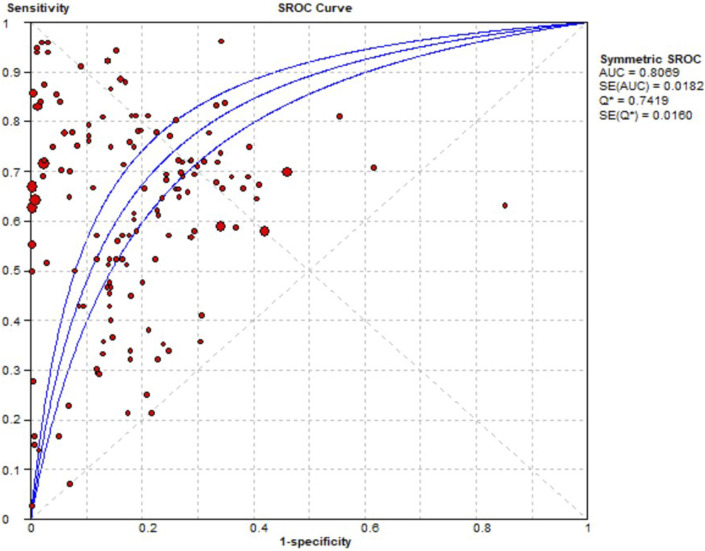
Pooled SROC of the prediction ADE.

Contingency tables from 33 prediction studies (Liu et al., 2018; Dong et al., 2019; Sun et al., 2020; Yoo et al., 2020; Herrin et al., 2021; Li et al., 2022; Okawa et al., 2022; Wu et al., 2022; Chen et al., 2023; Cheng et al., 2023; Gong et al., 2023; Goyal et al., 2023; Güven et al., 2023; Heo et al., 2023; Huang et al., 2023; Hu et al., 2023; Jeong et al., 2023; Jiang et al., 2023; Lee et al., 2023; Liao et al., 2023; Lu et al., 2023; Ma X. et al., 2023; Mao et al., 2023; Mora et al., 2023; Ruiz Sarrias et al., 2023; Zhou et al., 2023; Chiu et al., 2024; Lippenszky et al., 2024; Matsumoto et al., 2024; Nguyen et al., 2024; Patel et al., 2024; Surendran et al., 2024; Xiao et al., 2024) were extracted, which included 13 ADEs, 33 ML models, and 15 drugs. Significant heterogeneity was detected in the combined results for sensitivity (I^2^ = 99.1%, *p* = 0.00), specificity (I^2^ = 100%, *p* = 0.00), and DOR (I^2^ = 99.8%, *p* = 0.00). Consequently, a random effects model was used to evaluate the prediction accuracy of ADEs. The combined sensitivity, specificity, DOR, and AUC were 0.65 (95% CI: 0.65–0.66), 0.89 (95% CI: 0.89–0.90), 12.11 (95% CI: 8.17–17.95), and 0.8069, respectively. These results indicate that the prediction accuracy for ADEs is relatively robust. The AUC of the SROC curve is shown in Figure 4.

#### Subgroup analysis and heterogeneity

A subgroup analysis was conducted to assess the predictive performance of ML across different ADEs. The results indicated that ML demonstrated superior performance in detecting peripheral neuropathy and acute respiratory failure compared to other ADEs. Specifically, for peripheral neuropathy, the sensitivity was 0.85 (95% CI: 0.83–0.93), specificity was 0.75 (95% CI: 0.65–0.84), the DOR was 18.13 (95% CI: 7.05–46.63), and the AUC was 0.8794. For acute respiratory failure, sensitivity was 0.78 (95% CI: 0.72–0.84), specificity was 0.80 (95% CI: 0.78–0.81), the DOR was 13.00 (95% CI: 9.23–18.31), and AUC was 0.8561.

A subgroup analysis comparing the performance of different models showed that the pooled sensitivity for LR was moderate at 0.59 (95% CI: 0.58–0.59). The performance of GBM and GBDT appeared strong, with pooled sensitivities of 0.92 (95% CI: 0.88–0.95) and 0.81 (95% CI: 0.77–0.84), respectively. Heterogeneity decreased somewhat when analyzing the same ADEs or ML models, as shown in Supplementary Figures S2–S5 and Supplementary Table S6.

#### Publication bias

**FIGURE 5:**
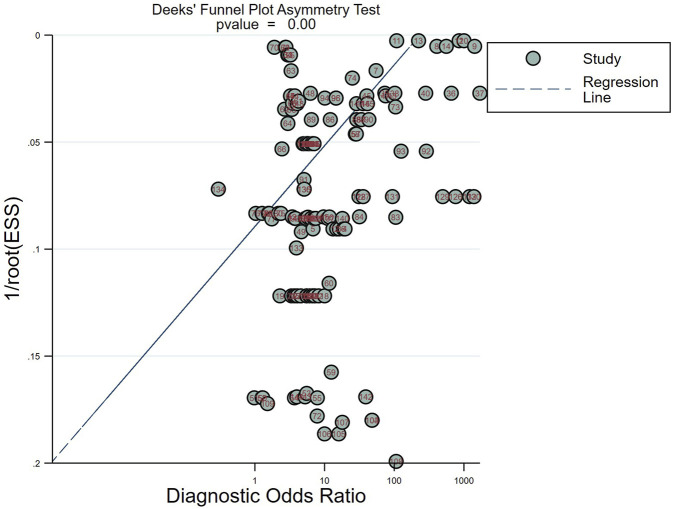
Deeks’ funnel plots for the assessment of potential bias in the meta-analysis for diagnosis.

The Deek funnel plot (Figure 5) was used to assess the presence of publication bias in the included studies. The results did not indicate significant publication bias in the studies included in this meta-analysis (*p* = 0.00).

## Discussion

We present the first comprehensive systematic review and meta-analysis assessing the performance of ML models in predicting ADEs based on EHRs. This review demonstrates that ML algorithms can complement traditional clinical decision-making in ADE prediction. The included studies were of high quality, and the applications of ML were diverse, covering a wide range of ADEs.

ML has been applied to predict ADEs for various drugs, with antitumor drugs being the most frequently studied. A total of 23 studies (15 on chemotherapy and 8 on tumor-targeting drugs) reported predictive outcomes related to ADEs associated with antitumor medications. Cancer remains the leading cause of death worldwide, and the necessity for anti-cancer drugs contributes significantly to the financial burden on individuals and healthcare systems. Cytotoxic chemotherapy remains the gold standard and first-line treatment for many common cancers (Schirrmacher, 2019). Chemotherapy-related drug toxicity may also occur more frequently than with other drugs, further increasing the economic burden (Livshits et al., 2014). Our findings indicated that renal dysfunction, cardiac events, and gastrointestinal events were the most reported ADEs in predictive model studies, aligning with the common ADEs associated with chemotherapy drugs. The excretion of chemotherapeutic metabolites through the urinary system can lead to nephrotoxicity, which varies depending on the type of chemotherapy, the malignancy being treated, the patient’s age, and the underlying conditions (Sahni et al., 2009). Many chemotherapy agents, particularly platinum coordination complexes, are known to cause kidney damage (Madias and Harrington, 1978). And nephrotoxicity has emerged as a significant limiting factor in the therapeutic use of these compounds. Among the included studies, multi-layer perceptron (MLP), ANN, and GBDT were used to develop prediction models for chemotherapy-related renal dysfunction, demonstrating good performance with AUC values ranging from 0.67 to 0.90 (Huang et al., 2022; Okawa et al., 2022; Chambers et al., 2023). Factors such as age, sex, body surface area, serum creatinine levels, and genetic predispositions may influence the occurrence of chemotherapy-related renal dysfunction (Huang et al., 2022; Okawa et al., 2022).

The included studies also frequently reported cardiotoxicity associated with chemotherapy, a life-threatening side effect (Chang et al., 2022; Li et al., 2022; Nguyen et al., 2024; Yagi et al., 2024). Anthracyclines were identified as the main agents responsible for chemotherapy-induced cardiotoxicity (Chang et al., 2022; Yagi et al., 2024). These drugs have been widely used and continue to serve as the cornerstone of treatment for multiple solid tumors and hematologic malignancies (Henriksen, 2018). The predictive capacity of the ML models for chemotherapy-related cardiac events showed AUC values ranging from 0.66 to 0.90 (Chang et al., 2022; Li et al., 2022; Nguyen et al., 2024; Yagi et al., 2024). The results indicated that, in addition to aging and male sex, pre-existing heart disease was a significant risk factor. This included conditions such as hypertension, reduced left ventricular ejection fraction, ischemic heart disease, arrhythmia, and coronary artery disease (Chang et al., 2022; Li et al., 2022; Nguyen et al., 2024; Yagi et al., 2024). In addition to cardiotoxicity, gastrointestinal events were frequently reported as side effects of chemotherapy. These events, including nausea, vomiting, ulcerative mucositis, and constipation, can significantly impact patient quality of life, although they are generally not life-threatening. The AUC values for ML in predicting gastrointestinal events were promising, with nausea and vomiting ranging from 0.81 to 0.85, ulcerative mucositis from 0.79 to 0.83, and constipation at 0.88 (Satheeshkumar et al., 2021; On et al., 2022; Zhang et al., 2023). Factors contributing to nausea, vomiting, and constipation included the number of treatment cycles, line of treatment, reduction in chemotherapy doses, chemotherapy regimens, and creatinine clearance (Satheeshkumar et al., 2021; On et al., 2022; Zhang et al., 2023). For ulcerative mucositis, strategies to mitigate risk included addressing conditions such as pancytopenia, agranulocytosis, fluid and electrolyte imbalances, and chemotherapy-induced anemia (Satheeshkumar et al., 2021).

Recently, novel targeted cancer therapies have emerged, designed to block biological transduction pathways and/or target specific cancer proteins to induce cancer cell death through apoptosis, immune system stimulation, or precisely delivering chemotherapeutic agents to cancer cells (Pérez-Herrero and Fernández-Medarde, 2015). Although targeted therapies reduce specific undesirable side effects compared to traditional chemotherapy, they can still lead to specific ADEs. Among the studies reviewed, immune checkpoint inhibitors (ICIs) were the most reported, with immune-related adverse events (irAEs) being the main associated ADE. Previous research indicates that irAEs affect 20%–30% of patients undergoing ICI monotherapy and more than 50% of those receiving combination ICI therapies (Martins et al., 2019; Gumusay et al., 2022). Pneumonitis was commonly reported in included studies, with occurrence rates exceeding 25%. The AUC of the ML prediction models ranged from 0.74 to 0.90 (Cheng et al., 2023; Gong et al., 2023; Lippenszky et al., 2024). Other irAEs associated with ICIs included hepatitis, colitis, thyroid disorders, cutaneous reactions, and cardiac irAEs, with occurrence rates around 10% and AUC values for ML prediction models ranging from 0.65 to 0.77 (Heilbroner et al., 2021; Kim W. et al., 2021; Lewinson et al., 2021; Lippenszky et al., 2024). These irAEs were particularly prevalent among patients with pre-existing underlying conditions, and an increase in inflammatory cell counts (lymphocytes, neutrophils, or eosinophils) was often observed in these patients (Heilbroner et al., 2021; Kim W. et al., 2021; Lewinson et al., 2021; Gong et al., 2023; Lippenszky et al., 2024).

Antibacterial agents are commonly used in inpatient settings, and approximately 50% of hospitalized patients receive at least one antibiotic during their stay (Tamma et al., 2017). Common antimicrobials-associated ADEs include gastrointestinal, renal, hepatic, and hematologic abnormalities (Tamma et al., 2017). Among the included studies, ML models were developed to predict hepatic (Asai et al., 2023; Ma J. et al., 2023) and renal (Imai et al., 2020; Mu et al., 2022; Chiu et al., 2024) dysfunction, thrombocytopenia (Patel et al., 2024; Zhao et al., 2024), anemia (Patel et al., 2024), and neutropenia (Imai et al., 2019). Antibacterial-related liver and kidney dysfunction were more likely to occur in patients with abnormal blood concentrations of the drugs or those with pre-existing liver and kidney diseases (Imai et al., 2020; Mu et al., 2022; Asai et al., 2023; Ma J. et al., 2023; Chiu et al., 2024). Additionally, antibacterial-related thrombocytopenia and anemia were associated with baseline platelet counts, international normalized ratio, hemoglobin levels, and overall liver and kidney function (Patel et al., 2024; Zhao et al., 2024). Ganciclovir-related neutropenia was common among patients with impaired liver and kidney function, those who received prolonged or high doses of ganciclovir, and individuals with low body weight (Imai et al., 2019).

Antituberculosis drugs, a specific class of antibacterial agents, were associated with ADEs in three of the included studies (Lai et al., 2020; Liao et al., 2023; Xiao et al., 2024). Antituberculosis drug-induced hepatotoxicity is a serious ADE linked to first-line tuberculosis medications such as rifampin, isoniazid, and pyrazinamide (Tostmann et al., 2008). This condition often requires the temporary discontinuation of these drugs to prevent progression to fulminant liver disease or even death. In predictive modeling, the AUC values for ML models that assess the risk of antituberculosis drug-induced hepatotoxicity ranged from 0.89 to 0.90, indicating high accuracy in predicting which patients may be at risk (Lai et al., 2020; Liao et al., 2023; Xiao et al., 2024). Patients with a history of abnormal liver function, underlying liver disease, and elevated liver enzymes had a higher risk of developing liver damage induced by antituberculosis drugs (Lai et al., 2020; Liao et al., 2023; Xiao et al., 2024). Genetic factors also played a significant role, and polymorphisms such as NAT27, OATP1B11a/1a, OATP1B11a/15, and UGT1A127/*28 contributed to the risk (Lai et al., 2020). This information can help clinicians better manage treatment plans and monitor patients more effectively to mitigate the risk of liver damage.

Five studies reported contrast-induced nephropathy (CIN), a common complication arising from intravascular use of contrast media during arterial angiography, with an incidence rate ranging from 3% to 15% (Sun et al., 2020; Ma X. et al., 2023; Yan et al., 2023; Zhou et al., 2023; Choi et al., 2024). The incidence of CIN continues to increase, making it the third leading cause of hospital-acquired acute renal injury (Mehran et al., 2004). While typically transient, CIN significantly increases the need for dialysis among affected patients, with a poor prognosis that often leads to prolonged hospital stays and elevated mortality rates (Subramanian et al., 2007; Ma X. et al., 2023). Commonly reported ML models to predict CIN include RF, XGBoost, GBDT, and SVM, with AUC values ranging from 0.72 to 0.94 (Sun et al., 2020; Ma X. et al., 2023; Yan et al., 2023; Zhou et al., 2023; Choi et al., 2024). CIN was more likely to occur in patients with pre-existing kidney dysfunction, including chronic kidney disease, elevated serum creatinine levels, impaired creatinine clearance, and abnormal blood pressure (Sun et al., 2020; Ma X. et al., 2023; Yan et al., 2023; Zhou et al., 2023; Choi et al., 2024).

The application of ML algorithms in predicting ADEs induced by various medications, including anti-inflammatory or opioid drugs, anticoagulants or thrombolytics, antituberculosis agents, thalidomide or lenalidomide, and methotrexate, showed promising results. The AUC for these ML prediction models typically ranged from 0.75 to 0.95, indicating strong performance in ADE prediction. A total of 38 different ML algorithms were identified in the studies reviewed, with ensemble learning techniques being the most prevalent. Ensemble learning combines predictions from multiple weak learners to enhance predictive accuracy (Ahn et al., 2023). The primary ensemble learning methods include the bagging and boosting algorithms. RF, a classic bagging algorithm, was the most frequently reported in 30 studies. RF aggregates predictions from multiple decision trees, each built from a bootstrapped version of the training data set (Hu and Szymczak, 2023). Among the studies using RF, seven identified it as the optimal model, with an average AUC of 0.770 ± 0.104 and a sensitivity of 0.630 ± 0.215. Unlike bagging, where each weak learner operates in parallel, boosting algorithms train weak learners sequentially, with each iteration focusing on misclassified instances from the previous round (González-Recio et al., 2013). Boosting algorithms appeared in 29 studies, including models such as XGBoost, LightGBM, GBDT, AdaBoost, and CatBoost. XGBoost was frequently highlighted as the optimal model, achieving an average AUC of 0.810 ± 0.109 and a sensitivity of 0.680 ± 0.212. Other ML models, such as SVM, ANN, and K-nearest neighbors (KNN), were also reported; however, their performance did not surpass that of RF and boosting algorithms. These findings show the effectiveness of ML algorithms, particularly ensemble methods, in predicting ADEs associated with various medications.

Class-imbalanced data introduce bias, particularly pronounced in high-dimensional datasets (Blagus and Lusa, 2013). T This issue can be mitigated through resampling methods that generate class-balanced data. The resampling methods reported include oversampling techniques such as the Synthetic Minority Oversampling Technique (SMOTE), SVM-SMOTE, undersampling with Tomek links (Tomek), and a combination of oversampling and undersampling methods using SMOTE and Tomek links (SMOTETL) (Chiu et al., 2024). Imbalanced data analysis was discussed in 23 studies, three of which specifically compared the performance of models with and without resampling methods (Lu et al., 2023; Chiu et al., 2024; Noda et al., 2024). The results indicated that the boosting algorithms demonstrated strong performance when combined with the imbalance analysis. The AUC values improved significantly, increasing from 0.601–0.936 to 0.786–0.939 (Lu et al., 2023; Chiu et al., 2024; Noda et al., 2024). Additionally, whether imbalance analysis was applied was one of the evaluation criteria on the ChAMAI checklist. The findings also indicated that feature selection could improve the model performance. Therefore, we recommend the application of imbalance analysis and feature selection in developing ADE prediction models to reduce bias and improve efficiency.

The quality of the included studies was assessed as high based on the AHRQ tool and the ChAMAI. According to the AHRQ tool, all items were considered equally important (AHQR, 2024). The results showed that the included studies often did not score on the items related to the control of confounding factors and follow-up procedures. In the ChAMAI, the 30 items were categorized into 10 low-priority and 20 high-priority items (Cabitza and Campagner, 2021). As a tool for evaluating the rigor of artificial intelligence methodologies, this tool places greater emphasis on the quality of the data processing procedures and the model-building process. Key aspects of data processing include outlier detection, handling missing values, feature pre-processing, and addressing data imbalance. The model-building process involves reporting tasks, specifying outputs, detailing model architecture, data splitting, training, selection, calibration, and validation procedures. Sharing code and data is also a high-priority item, as it enhances transparency and adaptability. While studies with high scores had good data processing procedures and model building, there remains room for improvement in code and data sharing.

## Limitations

This study has several limitations. First, although the overall quality of the included studies was high, there remains room for improvement. Specifically, items 8 and 11 of the AHRQ criteria received the lowest average scores, indicating the need for better control of confounding factors and better follow-up procedures. Additionally, the ChAMAI results suggested that improvements are needed in outlier detection and analysis and in code and data sharing. Furthermore, only two studies demonstrated the development and implementation of prediction systems in real-world applications (Jiang et al., 2023; Ruiz Sarrias et al., 2023). The limited practical application of these models may hinder both the studies’ quality and the models’ development. Second, significant heterogeneity was observed among the included studies. Although heterogeneity was somewhat reduced within the same ADE category, it was difficult to avoid completely. The variability was due to differences in the databases used, predictors, ML algorithms, hyperparameters, and populations studied, making it challenging to fully mitigate (Xie et al., 2022). Third, meta-regression was only conducted on the 33 studies that provided contingency tables, limiting our analysis. For the remaining studies, we only performed descriptive analyses of performance metrics. Fourth, we did not include studies that predict all ADEs in specific populations. We believe that the primary aim of these studies was to examine the epidemiology of ADEs within those populations, rather than to identify precise risk factors. Therefore, we recommend that these studies be comprehensively analyzed in a separate study. Finally, the included studies covered 15 different ADEs, 15 drug types, and 38 mL models, complicating the synthesis of results in these diverse studies.

## Conclusion

This systematic review and meta-analysis summarized the current research on using machine learning to predict ADEs, focusing on oncology-related treatments, including chemotherapy and tumor-targeting drugs. Among the ADEs analyzed, drug-related liver and kidney dysfunction was the most predicted due to its high incidence and potential to cause treatment interruptions. The study found that ML methods, particularly boosting algorithms such as XGBoost, GBM, and GBDT, effectively predict ADEs. Given the variability between studies, there is a need for more standardized research on different ADEs to ensure the accuracy and robustness of these predictive tools. Future research should prioritize multicenter studies incorporating diverse data types and evaluate the impact of artificial intelligence predictive models in real-world clinical settings.

## Data Availability

The original contributions presented in the study are included in the article/Supplementary Material, further inquiries can be directed to the corresponding authors.
