# Supplementary material for: Predicting adverse drug event using machine learning based on electronic health records: a systematic review and meta-analysis
Source: Front Pharmacol. 2024 Nov 13;15:1497397. doi: 10.3389/fphar.2024.1497397 (PMC11600142; doi:10.3389/fphar.2024.1497397)
Supplement: Supplementary file 1 [file DataSheet1.docx]

**Supplementary Table 1 Search Strategy**

| Database | Search Strategy |
| --- | --- |
| PubMed | (((machine learn [mh]) OR (machine learn [tw])) OR ((machine learning [mh]) OR (machine learning [tw])) OR ((deep learning [mh]) OR (deep learning [tw])) OR ((artificial intelligence [mh]) OR (artificial intelligence [tw])) OR ((artificial learning [mh]) OR (artificial learning [tw])) OR ((machine intelligence [mh]) OR (machine intelligence [tw])) OR ((neural networks [mh]) OR (neural networks [tw])))  AND (((adverse drug reaction [mh]) OR (adverse drug reaction [tw])) OR ((adverse drug event [mh]) OR (adverse drug event [tw])) OR ((ADE [mh]) OR (ADE [tw])) OR ((ADR [mh]) OR (ADR [tw])))  AND (((predict[mh]) OR (predict[tw])) OR ((prediction[mh]) OR (prediction [tw])) OR ((predicting[mh]) OR (predicting [tw])) OR ((risk [mh]) OR (risk [tw])) OR ((predictive [mh]) OR (predictive [tw]))) |
| Embase | (machine learn.mp. or exp machine learn/ or machine learning.mp. or exp machine learning/ or deep learning.mp. or exp deep learning/ or artificial intelligence.mp. or exp artificial intelligence/ or artificial learning.mp. or exp artificial learning/ or machine intelligence.mp. or exp machine intelligence/ or neural networks.mp. or exp neural networks/)  AND (exp adverse drug reaction/ or adverse drug reaction. mp. or exp adverse drug event/ or adverse drug event. mp. or exp ADE/ or ADE.mp. or exp ADR/ or ADR.mp.)  AND (exp predict/ or predict. mp. or exp predicting/ or predicting. mp. or exp prediction/ or prediction.mp. or exp risk/ or risk.mp. or exp predictive / or predictive.mp. or exp predictive/ or predictive.mp.) |
| Web of Science | TS= (machine learn OR machine learning OR deep learning OR artificial intelligence OR artificial learning OR machine intelligence OR neural networks)  AND TS= (adverse drug reaction OR adverse drug event OR ADE OR ADR)  AND TS= (predict OR prediction OR predicting OR risk OR predictive OR predictive) |
| IEEE | (machine learn OR machine learning OR deep learning OR artificial intelligence OR artificial learning OR machine intelligence OR neural networks) [Abstract]  AND (adverse drug reaction OR adverse drug event OR ADE OR ADR) [Abstract]  AND (predict OR prediction OR predicting OR risk OR predictive OR predictive) [Abstract] |

**Supplementary Table 2 Characteristics of included studies (Data period, Disease, and feature)**

| Classification of drugs | Study | Data period | Disease | Initial Features | Important features |
| --- | --- | --- | --- | --- | --- |
| Tumor immunotherapy | Lippenszky, 2024 | Up to 2018 | Cancer (Melanoma, Lung, Genitourinary) | Features classification: Patient Information, Cancer Diagnosis, Smoking History, PDL1 Status, MSI Status, Genomic Test Results, Treatment Information, Quality Control | Pneumonitis: lung codes, relative frequency of Malignant neoplasm of bronchus or lung code, smoking, Secondary malignant neoplasm of the respiratory or digestive organs, BMI, time-weighted average of oxygen saturation in blood values, Chronic obstructive pulmonary disease  Hepatitis: ALP, AST, T-BILI, ALT  Colitis: Anti-CTLA-4, chemotherapy, Malignant melanoma of the skin, ICI drug frequency, Low HGB, Low ALC, High WBC, Anti-PD-1, High ALC, High RBC, Low RBC. |
|  | Cheng, 2023 | 2016-2018 | Lung cancer | Age, Gender, Smoking, BMI, Normal weight, Overweight or obese, ECOG PS, Pre-existing lung diseases, Previous radiotherapy, Histology, TNM stage, ICI type, ALC, NLR, PLR, LMR, Albumin, LDH, CT score | Unfiltered |
|  | Gong, 2023 | 2010-2021 | Lung cancer | Features classification: demographic, treatment, laboratory results | Number of underlying diseases >=2, History of lung diseases, Sindillizumab, NSCLC, Tirelizumab, KPS score ≤70, Cancer stage, Percentage of CD4+ lymphocyte, History of antitumor therapy, HGB, Body temperature |
|  | Heilbroner, 2021 | N | Cancer (Melanoma, Lung, renal cell carcinoma) | Specific features not mentioned | HR, Cl, Weight, Age, Neut%, Corticosteroid, Serotonin-3 Receptor Antagonist, T, Phenothiazine, Lymphocyte Percent, Microtubule inhibitor, BUN,BMI, Mg, PT, DBP, Hb, Eosinophils Percent, PLT, AST, SCr, ALC, Folate Analog Metabolic Inhibitor, Chronic Pulmonary Disease, LDH, Substance P/Neurokinin-l Receptor Antagonist, Male, Absolute Neut#, Stage 4,Osmotic Laxative, Performance Status, Na, SBP, Charlson Score, renal cell carcinoma, Angiotensin Converting Enzyme Inhibitor, Female, TBILI, Delta Weight, DBILI |
|  | Jiang, 2023 | 2014-2021 | B-cell lymphomas | Gender, Age ,Weight , BMI , BSA, Diagnosis, Courses of previous antineoplastic treatment, Antineoplastic agents combined, Zanubrutinib, Scr , Ccr , ALT , AST , ALP, TP , ALB, TBIL, LDH, Neut#, HGB, PLT, ALC , WBC , RBC , Fib , PT , Number of comorbidities, Co-administration of antihypertensive drugs , Co-administration of hypoglycemic agents , Co-administration of statins, Co-administration of antibacterial agents, Co-administration of antifungal agents, Co-administration of antiviral agents, Co-administration of antiplatelet and anticoagulant medications , Co-administration of PPI or H2RA, Co-administration of CYP3A inhibitors | WBC, LDH, TP, Fib, RBC, AST, BTKI, Neut#, Gender, PLT |
|  | Kim,2021a | 2015-021 | Cancer | Gender, Age, BMI, Heart disease, CYP2Y12 inhibitors, Smoking history, Hypertension, Opioids, B-blockers, BMI | Gender, Age, BMI, Heart disease, CYP2Y12 inhibitors, Smoking history, Hypertension, Opioids, B-blockers, BMI |
|  | Lewinson, 2021 | N | Cancer (Melanoma, Lung) | Tumor type, Treatment drug, Age, Autoimmune history, Derived NLR, LDH, Albumin, BMI, ECOG PS, TNM stage | Unfiltered |
|  | Kim,2021b | 2015-2020 | hematologic malignancies and neurodegenerative disorders | Gender, Age, Weight, Height, BSA, Alcohol history, CVD or DM, Daily dose, Anticancer drugs, Anticancer drugs, HMG-coA reductase inhibitors, Antihypertensives, Immunosuppressants, Immunosuppressants, CYP3A4 inducers, H2 blockers, PPIs | Male, Age, CVD or DM, Alcohol history, Daily dose, Immunosuppressants, H2 blockers |
| Low-Dose MTX (for immune disease) | Surendran, 2024 | 2016-2018 | RA | Age, Gender, NAFLD, HGB, Total count, Neut%, ALC percentage, NLR ratio, AST, ALT, SCr, Duration of MTX therapy, Initial MTX dose, Initial MTX dose class, Maximal MTX dose, Maximal MTX dose class, MTX monotherapy, MTX plus sulfasalazine, MTX plus leflunomide, MTX plus mycophenolate, MTX plus hydroxychloroquine, Triple regimen | Total White Blood Cell Count, HGB level, Duration of MTX therapy, Age, NLR Ratio, SCr, Neut%, Lymphocyte Percentage, Baseline AST, Baseline AST |
|  | Hu, 2023 | 2020-2021 | Immune system disorders | Number of comorbidities, Number of drugs, BMI, Antibiotic usage, Age, Gender, Doses of folic acid, Other immunosuppressive agent usage, First MTX use, NSAIDS, Alcohol usage, DM, Chinese patent medicine usage, Infectious liver disease, Dyslipidemia, History of kidney disease, Glucocorticoid use | BMI, Age, Number of drugs, Number of comorbidities, Dyslipidemia, Antibiotic usage, NSAIDS, Gender, Other immunosuppressive agent usage, Glucocorticoid use, First MTX use |
| Chemotherapy | Chambers, 2023 | 2013-2018 | Cancer (Breast, diffuse large B-cell lymphoma, Colorectal) | Not mentioned | Not mentioned |
|  | Chang, 2022 | 2014-2018 | Breast cancer | Herceptin, hypertension, dose, BMI, operation, age, 5-FU, LVEF, radiotherapy, Caner stage, Taxanes, DM, coronary artery disease, Doxorubicin or epirubicin, cyclophosphamide | Herceptin, hypertension, dose, BMI, operation, age, 5FU, left ventricular ejection fraction, radiotherapy, Caner stage |
|  | Huang, 2022 | 2005-2011 | Non-small cell lung cancer | Clinical and genomic features | Age, genomic features (MATE1_1, CT, XPA, NQO, MATE1_2, GSTP1) |
|  | On, 2022 | 2015-2016 | Cancer (Colorectal cancer, Gastric cancer, Breast cancer, Hepatobiliary and pancreatic cancer, Lung cancer, Urinary tract cancer, Colorectal and Gastric cancer) | Features classification: Demographics, Antineoplastic therapy related features, Type of chemotherapy, Type of cancer, Previous history of ADRs, Comorbidity | Age, Gender , Number of cycles, Line of treatment, Chemotherapy dose reduction, Combined targeted therapy, Combined radiotherapy, FOLFOX, FOLFIRI, Paclitaxel, Colorectal cancer, Lung cancer, Gastric cancer, Hepatobiliary-pancreatic cancer, Secondary cancer ,Previous history of NV, Previous history of FA , Previous history of DI , Previous history of PN , Previous history of HS , Previous history of ST, Previous history of HFS, Bone marrow depression, DM, Lung disease, Malnutrition, Infection ,Heart disease ,Liver failure, Kidney failure (Different ADES have different influencing factors, see the original article for details) |
|  | Sarrias, 2023 | 2010-2020 | Gastrointestinal Cancer | Features classification: Age, Gender, ECOG PS, Histology, Origin, Stage, Chemotherapy, Pyrimidines Metabolism, Patient Status, BMI, blood concentration, Cell Blood Count, Renal function | Top ten: the difference in days between the day of the AUC2 measurement and the day of the AUC1 measurement, Chemotherapy, AUC-1, Origin, the difference in days between the day of the AUC2 measurement and the day of the first analytical measurement, eGFR, AUC-2, Eosinophils, Platelet amplitude distribution, Platelet |
|  | Satheeshkumar, 2021 | 2017 | Cancer (unspecified) | Not mentioned | Pancytopenia, agranulocytosis, fluid and electrolyte imbalance, age, anemia due to chemotherapy, median household income based on zip code, and depression |
|  | Venäläinen，2021 | 2010-2017 | Cancer (Breast, Central nervous system, Colorectal, Female reproductive, Gastric, Head and neck, Lung, non-small cell, Lung, small cell, Melanoma, Other gastrointestinal, Pancreas and gallbladder, Prostate, Sarcoma, Testicular, Urinary tract, Other) | Features classification: Demographics, Metastatic disease, Planned relative dose intensity, Intravenous treatment regimens, Cancer group | Breast cancer, Sarcoma, Neut#, Thrombocyte count, Use of taxanes, Combined use of taxanes and monoclonal antibodies, Use of topoisomerase inhibitors, Use of antimetabolites, Use of granulocyte colony-stimulating factor, Relative dose intensity <85% |
|  | Zhang,2023 | 2018-2021 | Cancer (Lung, Breast, Gynecologic, Gastrointestinal, Esophageal, Others) | CCr, Age, Gender, Anticipatory NV, Antiemetic regimen, Nausea or vomiting in the prior cycle, highly emetogenic drug, Cycle number, Drinking, ALT, TBILI, AST, GLB, DBILI, ALB, TP, Use of non-prescribed antiemetics at home, Sleep less than 7 hours, Morning sickness | Ccr, age, gender, anticipatory NV, and antiemetic regimen |
|  | Zhou,2021 | 2016-2020 | Colon or rectal cancer | Age, BMI, Colostomy, Complications, cancer related anemia, Depression, DM, Total score_QLQ-C30, Exercise, Hypercholesterolemia, Diet, Marital status, Education level, Pathological stage | Age, BMI, Colostomy, Complications, cancer related anemia |
|  | Li, 2022 | 2006-2014 | Colorectal cancer | Specific features not mentioned | Pre-existing ischemia heart disease diseases, Pre-existing arrhythmia, Pre-existing heart failure, Pre-existing endocarditis, Prior anticoagulant medication, Patient age at using fluoropyrimidine, Prior beta-blocker medication, Pre-existing cardiomyopathy, County-level graduation rates of high school or above, Pre-existing hypertension, County-level median household income, Pre-existing IHD diseases, Tumor sizes, Pre-existing other symptoms involving cardiovascular system, Pre-existing heart failure, White race, Prior surgery, Prior antiplatelet medication |
|  | Matsumoto, 2024 | 2012-2021 | Urothelial Cancer | Specific features not mentioned | PLT, HGB, ALC, and dose of gemcitabine |
|  | Huang,2023 | 2016 | Cancer | Age, BMI, Chemotherapy regimens, Weight, Single dose of Oxaliplatin, Total dose of Oxaliplatin, Height, Glucocorticoid drugs, Proton pump inhibitors, Chemotherapy cycles, Liver protective drugs, 5-HT3 receptor antagonists, Cerebral infarction, Gastritis, KPS score, Antihistamine drugs, History of hepatitis B, Duodenal ulcer, Gender, DM, Hypertension, History of renal calculi | Age, chemotherapy regimen, number of chemotherapy cycles, single dose of Oxaliplatin, total dose of Oxaliplatin, glucocorticoid drugs, and antihistamine drugs |
|  | Nguyen,2023 | 2004-2020 | Breast Cancer | Features classification: demographics, cancer conditions, comorbidities, concurrent medications, laboratory test | Unfiltered |
|  | Yagi,2024 | N | Cancer (Lymphoma, Leukemia, Breast cancer, Sarcoma, Other) | Not mentioned | Age, Gender, race, cancer types, low baseline observed LVEF on echocardiogram, comorbidities such as coronary artery disease and hypertension, and the presence of overt ECG abnormalities |
|  | Okawa, 2022 | 2006-2013 | Not mentioned | Gender, Age, Body surface area, SCr, Serum albumin, History of DM, History of Cardio vascular disease, Maximum daily cisplatin dose | Gender, Age, Body surface area, SCr, Serum albumin, History of DM, History of Cardio vascular disease, Maximum daily cisplatin dose |
| Antibacterial agents | Imai, 2019 | 2008-2018 | Organ transplantations, Hematopoietic stem cell transplantations, Hematological malignancies, Acquired immunodeficiency syndrome, Autoimmune diseases, Others | Features classification: Age, Gender, Weight, Neut#, PLT, SCr, Ccr, With hemodialysis, TBILI, ALB, Overdosage of initial ganciclovir dose, Total ganciclovir dose, Duration of therapy, Concomitant medications, Underlying diseases, Residence in intensive care unit, cytomegalovirus antigenemia | Unfiltered |
|  | Mu,2022 | 2015-2020 | Cancer, DM, Hypertension, Haptic insufficiency, Pancreatitis, Shock, Heart Failure | Features classification: Basic information, Vancomycin administration, Concomitant diseases, Concomitant medications, Laboratory Indicators | CRE, ALT, cystatin-C, Diuretics, blood urea, GGT, PLT, Human albumin, Ccr, BUN, Neut%, ALP, AST, Trough level, Neut#, ALB, monocyte count, Age, TBIL, Weight |
|  | Imai,2020 | 2011-2019 | Not mentioned | Trough concentration, Piperacillin-Tazobactam use, Vasopressor drugs use, Amphotericin B use, Duration of therapy, Furosemide use, Ccr, Residence in ICU | Trough concentration, Piperacillin–Tazobactam use, Vasopressor drugs use, Amphotericin B use, Duration of therapy |
|  | Zhao,2024 | 2011-2021 | Not mentioned | Gender, Age, Heigh, Weight, BMI, History of hypertension, History of DM, History of malignancy, Route of administration, Combined use of UFH, Total days on drug, Baseline PLT, TP, ALB, ALT, AST, TBIL, DBIL, Urea, Ccr | Age, History of hypertension, History of malignancy, Baseline PLT, TP, ALB, AST, DBIL, Urea, Ccr |
|  | Patel,2024 | 2012-2023 | Vancomycin-resistant Enterococcus faecium infection, Methicillin-resistant Staphylococcus aureus infection, Sepsis, Bacterial pneumonia, Bacteremia, Endocarditis, Infection of skin and/or subcutaneous tissue, Infection of bone and/or joint, Bacterial meningitis, Urinary tract infection, Tuberculosis or other mycobacteria, Other or unspecified bacterial infection | Features classification: Linezolid exposure, Demographics, Measurements, Conditions, Medications and procedures | Thrombocytopenia: PLT count, TBILI, INR, Absolute Neut#, AST, SCr, eGFR, Blood urea nitrogen, ALT, NLR, HGB, Weight, age, hepatic failure, ALB, Height Anemia: PLT count, HGB, TBILI, Urea, sCr, INR, Absolute Neut#, ALB, Age, eGFR, AST, Weight, NLR, ALT, Height, ICU |
|  | Asai, 2023 | 2019-2022 | Infection | Gender, Age, BMI, eGFR, Daily dose, BUN, ALT, AST, ALP, ALBI score, CRP, Concomitant drugs, Site of infection | Unfiltered |
|  | Ma,2023a | 2008-2020 | Infection | Not mentioned | Not mentioned |
|  | Chiu, 2022 | 2011-2020 | Not mentioned | Features classification: demographics, Colistin dosing information, Infection site, Pathogen, Comorbidity, Concurrent nephrotoxins, Chronic nephrotoxin use, Baseline laboratory results, Latest laboratory results, Laboratory value trends | Days of Medication Use, Colistin base activity cumulative dose, Colistin base activity daily dose, Latest CRP, Baseline HGB, slope of SCr, BMI, sepsis, CKD, slope of eGFR, Latest BUN, Colistin base activity dose per day per kg, slope of Neut#, Baseline K, Baseline Ccr, Latest WBC, Latest Na, Baseline WBC, slope of BUN, weight |
| Antituberculotic | Lai,2020 | N | Pulmonary tuberculosis | Gender, Age, Weight, Comorbidities, Hypertension, DM, Asthma, COPD, GI-related diseases, Renal diseases, Smoking, Alcoholics, Baseline liver function, AST, ALT, Rifampin, Isoniazid, Pyrazinamide, Genomic risk factors: NAT2*7, OATP1B1*1a/1a, OATP1B1*1a/15, UGT1A1*27/*28 | Age, Gender, weight, ALT, AST, smoking, drinking, Genomic risk factors: NAT2*7, OATP1B1*1a/1a, OATP1B1*1a/15, UGT1A1*27/*28 |
|  | Xiao,2024 | 2015-2020 | TB | Specific features not mentioned | Outpatient drug-induced liver injury, outpatient drug induced hepatitis once occurring, outpatient drug induced hepatitis sporadically occurring, inpatient drug induced hepatitis once occurring, outpatient drug induced liver injury sporadically occurring, inpatient drug induced hepatitis frequently occurring, inpatient drug induced liver injury once occurring, outpatient drug induced liver injury frequently occurring, TBILI, ALP, inpatient drug induced liver injury sporadically occurring, ALT, fatty liver disease, education, age |
|  | Liao,2023 | 2004-2021 | TB | Features classification: Gender, age, TB type, disease history, TB medication, and laboratory data | Unfiltered |
| Anti-inflammatory or Opioid | Jeong, 2023 | 2009-2013 | DM, Dyslipidemia, Angina pectoris, Myocardial infarction, Stroke, End stage renal disease, Chronic obstructive pulmonary disease, Cirrhosis, Hypertension, Gastroesophageal reflux disease, Acute upper respiratory infection, Mental and behavioral disorders | 12 comorbidities and 26 types of NSAID | Acute upper respiratory tract infection, Gastroesophageal reflux disease, Aceclofenac, Loxoprofen, Talniflumate, Meloxicam, Dexibuprofen |
|  | Lee,2022 | 2016–2017,2019 | Atrial fibrillation, Congestive heart failure, COPD, Cancer, Dementia, DM, Gastroesophageal reflux disease, Crohn's disease, Hypertension, Hyperlipidemia, Liver disease, Alcoholism, Renal disease, Peripheral vascular disease, Cerebral vascular disease, HIV infection | Features classification: Age, Gender, High dose of NSAIDs, Type of NSAIDs, Concomitant antithrombotic age, Concomitant glucocorticoid, Concomitant selective serotonin inhibitor, Concomitant selective serotonin inhibitor, Concomitant bisphosphonate, History of GI bleeding or perforation, Severe renal disease, Liver cirrhosis, Cancer | high-dose NSAIDs, non-selective NSAID, complicated GI ulcer history, male Gender, concomitant gastroprotective agents, relevant co-medications, severe renal disease and cirrhosis |
|  | Liu, 2018 | 2004-2006 | Osteoarthritis | Features classification: Demographics, Anthropometry, Comorbidity, Blood Measures and Physical activity Measures | Age, Radial pulse, Asthma, Weight, BMI, Height, Physical activity scale, Repeated chair stands time1, Operation to unclog or bypass arteries in legs, Diastolic, Mental summary scale, Systolic, walk pace,20-meter walk steps1, Repeated chair stands time2, DM, Physical summary scale,20-meter walk steps2, 20-meter walk time, Heavy housework |
|  | Dong,2020 | SPARCS: 2005-2016, Health Facts database: 2000-2017 | Not mentioned | Specific features not mentioned | SPARCS: Sedative, hypnotic or anxiolytic dependence, continuous, Unspecified viral hepatitis C without hepatic coma, Other alteration of consciousness, Cannabis abuse, unspecified, Anxiety state, unspecified, Unspecified drug dependence, unspecified, Sedative, hypnotic or anxiolytic abuse, unspecified, Toxic effect of ethyl alcohol, Other chronic pain, Pedestrian injured in collision with two- or three-wheeled motor vehicle, Altered mental status, Other, mixed, or unspecified drug abuse unspecified, Chronic pain syndrome, Acute respiratory failure, Lumbago, Poisoning by other specified central nervous system stimulants, Depressive disorder, not elsewhere classified, Poisoning by benzodiazepine-based tranquilizers, Tobacco use disorder, Deep necrosis of underlying tissues with loss of a body part, of multiple specified sites HF: Pulse, HR, Advanced Directive, Under New or Established Patient Emergency Department Services, Pulse Posterior Tibial Right, Tobacco Use, Sodium Chloride, Respiratory Rate, Other, mixed, or unspecified drug abuse, unspecified, Triage Notes, Essential hypertension, Pain Scale Score, Temperature, History of Fall, Pain Symptom Date of Onset, BMI, Pulse Dorsalis Pedis Left, Emergency department visit for the evaluation and management of a patient, Tobacco use disorder, Tobacco use disorder |
| Anticoagulation or thrombolysis | Xu, 2022 | 2016-2021 | Hypertension, DM, Coronary artery disease, Atrial fibrillation | Features classification: Age, Weight, Gender, Past history, Comorbidities, Baseline laboratory test, Laboratory test on the second day after IVT | Sodium, NLR, Calcium, AKP, BUN, RBC, PT, ALC, WB, INR, SBP on admission, HGB, Baseline NIHSS, lipoprotein, triglycerides |
|  | Mora,2023 | study population: 2010-2020, prospective validation: 2020-2021 | Peripheral artery disease, Arterial hypertension, Esophagitis, Hiatal hernia, Esophageal varicosities,  Gastroduodenal ulcer, Gastric erosions, Ulcerative colitis, Crohn's disease, Angiodysplasia, Liver cirrhosis, Chronic liver disease, Dementia | Gender, Age, Weight, Hospitalized vs. outpatient diagnosis, HR, SBP, Concomitant therapy with NSAIDs at baseline, Concomitant therapy with antiplatelets at baseline, Hospitalization required due to the index VTE event, Recent major bleeding, Prior myocardial infarction, Prior ischemic stroke, Peripheral artery disease, History of hypertension, Esophagitis, Hiatal hernia, Esophageal varices, Gastroduodenal ulcer, Gastric erosions, Ulcerative colitis, Crohn´s disease, Angiodysplasia, Liver cirrhosis, Chronic liver disease, Dementia, Heavy alcohol intake, Hemoptysis, Abnormal mental status, Right ventricular hypokinesia, Lower limbs thrombus proximity, Active Cancer, Recent surgery, Recent immobilization, Prior DVT or PE, Hormonal treatment al baseline, History of birth, HGB levels, Leukocyte count, PLT count, D-Dimer levels, Abnormal PT, D-Dimer value , Thrombophilia, CRE, Cava filter , Symptomatic PE, Concomitant therapy with corticosteroids at baseline, Syncope, Initial therapy with low molecular weight heparin, Initial therapy with UFH, Initial therapy with fondaparinux, Initial therapy with thrombolytics, Initial therapy with direct oral anticoagulants, Initial therapy with vitamin K antagonists, Initial therapy with others. | Baseline HGB, Age, Active cancer, DOACS, Abnormal mental status, Recent bleeding, Steroids, HR, Thrombolytics, Hypertension, Weight, BP, Antiplatelets, CRE, Syncope, Leukocytes, Gender, Diagnosis during hospitalization, Prolonged PT, D-Dimer, Prior myocardial infarction, Recent immobilization, Symptomatic PE, Liver cirrhosis, Thrombophilia, Hospitalized due to VTE, Gastroduodenal ulcer, Initial therapy with UFH, NSADs at baseline, Dementia, Gastric erosions, Hiatal hernia PLT, Esophageal varicosities |
|  | Chen, 2023 | 2021-2023 | Not mentioned | Gender, Age, BMI, Rivaroxaban dose, Antiplatelet drugs, Hypertension, DM, High triglyceride, High cholesterol, LDL-cholesterol abnormal, Lowest HGB, Lowest PLT, Coronary disease, Heart failure, valvopathy, PCI, apoplexy, Hemorrhage history, Coagulopathy, TT, APTT, INR, D-dimer, ALT, AST, BUN, CRE | Lowest PLT, BMI, APTT, TT, D-dimer, Lowest HGB, CRE, INR, ALT |
|  | Herrin,2021 | 2016-2019 | DM, Hypertension, Peripheral arterial disease, Alcoholism, Chronic kidney failure, Chronic liver disease, Rheumatologic disease, Carotid revascularization, Helicobacter pylori infection, History of GI bleeding, Smoking, Sleep apnea, Thyroid disease, Valvular heart disease, Viral hepatitis, Percutaneous coronary intervention, Charlson comorbidities | Features classification: Age, Race/ethnicity, Gender, Condition group, Baseline comorbidities and medications | Prior gastrointestinal bleeding, atrial fibrillation, ischemic heart disease, and venous thromboembolism, proton pump inhibitor/histamine 2 blocker, ischemic heart disease and venous thromboembolism, chronic renal failure, Hypertension, Female, Smoking, atrial fibrillation and ischemic heart disease, Alcohol use, indicates anticoagulants and antiplatelets treatment, Chronic liver disease, helicobacter pylori, Prior PCI, peripheral arterial disease, Diabetes with insulin, SSRI, Rheumatism, Dementia, White, Viral hepatitis, Antilipids, Sleep disorder, atrial fibrillation, Valvular heart disease, Age, Black, Male |
| Contrast agent | Choi,2024 | 1994-2021 | Heart valve disorder, Type 2 DM | Not mentioned | Chronic kidney disease, Hematocrit, Urea nitrogen, Natriuretic peptide, B prohormone N-Terminal, Troponin l cardiac, Base excess, Age |
|  | Zhou,2023 | 2016-2020 | Not mentioned | Specific features not mentioned | Age, Hb concentration, gender, NLR, Systolic blood pressure, FPG, CKD, Spironolactone, Low molecular weight heparin, eGFR, SCr, loop diuretic, β-blocker |
|  | Sun, 2020 | 2012-2018 | Hypertension, DM, Hyperlipidemia, Atrial fibrillation, Coronary heart disease, Heart failure, Stroke, CKD, COPD, Chronic liver diseases | Not mentioned | Neut%, Age, Free triiodothyronine, Preoperational hypotension, SCr, LDL cholesterol, HGB, Total triglycerides, Cardiac troponin I, white blood cell, High-density lipoprotein cholesterol, Brain natriuretic peptide, HR, LVEF, DBP, BMI, SBP, Total cholesterol, Weight, HbA1c |
|  | Yan, 2023 | 2015-2020 | DM, DM complication, Renal stone, Congestive heart failure, Cerebrovascular disease, Hypertension, Renal carcinoma, Sepsis | Neut#, eGFR, PLT count, Serum calcium, Serum sodium, BUN, Diuretics, Proteinuria, Renal CTA, Mechanical ventilation, Serum albumin, Total protein, TBIL, Antimicrobial drugs | Neut#, eGFR and PLT |
|  | Ma, 2023b | 2017-2020 | Hypertension, DM, Vascular diseases | Features classification: demographics, laboratory results, Physical examination results | HGB, DM, peripheral artery disease, age, uric acid, cystatin-C level, Creatine kinase isoenzyme MB level, systemic immune-inflammation index, TP, LDL and N terminal pro B type natriuretic peptide |
| Thalidomide or lenalidomide | Mao, 2023 | 2016-2022 | Crohn’s disease | 18 baseline variables and 150 genetic variables | Females, Age, Gender, Daily dose, ALT, AST, Hb, BUN, CRE, Neut#, PLT, HCT, CRP, WBC, LP, HDL-c, LDL-c, TG, Genes |
|  | Maray, 2023 | 2014-2018 | Cancer (unspecified) | Age, Gender, Comorbidities, Indication, Initial dose, Cytogenetics, Albumin corrected calcium corrected, Renal clearance, HGB, Bone involvement, chylomicron in urine, Monoclonal peak, PLT, LDH, Neut#, Albumin, Albumin, Revised International Staging System. | Unfiltered |
|  | Yoo,2020 | N | Attention deficit hyperactivity disorder | Features classification: demographics, clinical variables and genetic/environmental variables | Unfiltered |
| Other | Lu, 2023 | 2013-2017 | Hypertension, Bradycardia, DM, Anemia, Gout, Chronic renal failure, Renal dysfunction | Cumulative dose, Duration, PDD, AGE, Therapeutic days, BMI, Cholesterol, gastrin-releasing peptide, TSH, FT4, ALT, hematocrit, mean corpuscular HGB, HDL, LDL, alkaline phosphatase, Cholesterol, AST, SCr slope, ALT slope, Gender, average dose, NSAIDs, allopurinol, anti-DM, metformin, arrhythmia type, bradycardias, anemias, hypertensions, DMs, DM nephropathy, DM, renal dysfunction, T3 REMARK, SCr REMARK, Cholesterol REMARK, HDL REMARK, alkaline phosphatase REMARK, RBC, HGB, Mean Corpuscular Volume, mean corpuscular-HGB concentration, triglyceride, SCr, AST slope, Cholesterol slope | TSH, FT4, Duration, alkaline phosphatase, HDL, Total days of use, Cumulative dose, LDL, AGE, mean corpuscular HGB, SCr, ALT, hematocrit, AST, BMI, Cholesterol, gastrin-releasing peptide, tuberculin test, ALT slope |
|  | Simon, 2021 | 2003-2018 | Hypertension, Coronary artery disease, Heart failure, DM, Obesity, Chronic kidney disease | Age, Gender, Hypertension, Coronary artery disease, Heart failure,  DM, Obesity, Chronic kidney disease | Unfiltered |
|  | Wu, 2022 | 2010-2018 | N | Features classification: demographics, laboratory results, Physical examination results | Pre-treatment serum levels, Renal function, Dermatoses, Gender, Age, Dose, LDL, Hypoproteinemia, Anti-infective agents, Pre-treatment indicators of carcinoma, HGB, History of allergy, Respiratory diseases, Respiratory diseases, RBC, CRP, Dermatology medication, Eosinophils, Charlson comorbidity index (Score), Dermatology medication |
|  | Goyal,2023 | 2018 | Hypertension, renal disease, liver disease, cerebrovascular disease, bleeding disorder, organ transplant | Features classification: Demographics, Race/ethnicity, Comorbidities, Bleeding history, Socioeconomic, Alcohol use, Smoking, Recreational drug use, Concurrent drug use: antithrombotic, Concurrent drug use: non-steroidal anti-inflammatory drugs (NSAIDs), Concurrent drug use: glucocorticoids, Health literacy, Current SSRl use, SSRl used just before the newly prescribed SSRI, Number of prior SSRl switches | The specific results of feature selection were not shown |
|  | Heo,2023 | 1994-2021 | Not mentioned | Features classification: demographics, laboratory results, Physical examination results, drugs | Lymphocytes, Albumin, Protein, Eosinophils, Cholesterol, PT, Monocytes, Hypertensive disorder, 100 ML sodium chloride 9 MG/ML Injection, Calcium |
|  | Güven,2023 | 2020-2021 | DM, Uncontrolled DM, Coronary Artery Disease, Heart Failure, Chronic Kidney Disease, Obstructive Airway Disease, Active Cancer, Connective tissue Disease | Specific features not mentioned | Index K, Uncontrolled DM, Index Glucose, Chronic Kidney Disease, B-blocker Use, Insulin Use, NSAID Use, Index Uric Acid, β2 Agonist Use, Age |
|  | Noda,2023 | 2012-2020 | Ulcerative colitis, interstitial pneumonia associated with dermatomyositis or polymyositis | Age, Height, Weight, Neut#, ALC, PLT count, HGB, mean corpuscular HGB, CRP, SCr, BUN, Albumin, Aspartate aminotransferase, Alanine aminotransferase, LDH, Na, K, The number of varieties of nephrotoxic medicines at TAC initiation, Cumulative number of varieties of concomitant nephrotoxic medicines before the occurrence of nephrotoxicity, TAC concentration, Gender, ulcerative colitis, interstitial pneumonia associated with dermatomyositis or polymyositis | Unfiltered |

PDL1, Programmed cell death 1 ligand 1, MSI status, microsatellite-instability status, ECOG PS, Eastern Cooperative Oncology Group Performance Status, TNM stage, Tumor Node Metastasis, ALC, absolute lymphocyte count, NLR, neutrophil/lymphocyte ratio, PLR, platelet/lymphocyte ratio, LMR, lymphocyte/monocyte ratio, LDH, lactate dehydrogenase, CT, computed tomography, KPS, Karnofsky performance status, NSCLC, non-small cell lung cancer, HR, heart rate, BUN, blood urea nitrogen, BMI, Body Mass Index, PT, Prothrombin time, BP, blood pressure, DBP, diastolic blood pressure, SBP, systolic blood pressure, SCr, serum creatinine, Ccr, creatinine clearance rate, ALT, Alanine aminotransferase, AST, Aspartate aminotransferase, ALP, Alkaline phosphatase, TP, Total protein, ALB, Albumin, TBIL, Total bilirubin, LDH, Lactate dehydrogenase, Neut#, Neutrophil count, Neut%, Neutrophil percent, HGB, Hemoglobin concentration, PLT, Platelet count, WBC, White blood cell count, RBC, Red blood cell count, Fib, Fibrinogen, PPI, Proton pump inhibitor, H2RA, H2 Receptor Antagonist, CYP, Cytochrome P450, CVD, Cardiovascular disease, DM, diabetes mellitus, HMG-coA, hydroxy-beta-methylglutarate monoacyl-coA, NAFLD, non-alcoholic fatty liver disease, NSAIDS, Non-Steroidal Anti-inflammatory Drugs, LVEF, left ventricular ejection fraction, FOLFOX, Oxaliplatin+Calcium folinate+5-fluorouracil, FOLFIRI, Irinotecan+Calcium folinate+5-fluorouracil, TBILI, total bilirubin, DBILI, direct bilirubin, GLB, globulin, TP, total protein, IHD, Ischemic Heart Disease, 5-HT3, 5-hydroxytryptamine 3, ICU, intensive care unit, eGFR, estimated glomerular filtration rate, CRP, C-reactive protein, TB, tuberculosis, HIV, human immunodeficiency virus, GI, gastrointestinal, IVT, intravenous thrombolysis, RBC, Red blood cell, AKP, alkaline phosphatase, WB, white blood cell, COPD, chronic obstructive pulmonary disease, INR, international normalized ratio, NIHSS, the National Institutes of Health Stroke Scale, VTE, Venous thromboembolism events, DVT, Deep vein thrombosis, PE, pulmonary embolism, PCI, percutaneous coronary intervention, HDL , high density lipoprotein, LDL, Low Density Lipoprotein, APTT, activated partial thromboplastin time, TT, thromboplastin time, HbA1c, glycosylated hemoglobin, CTA, CT angiography, UFH, unfractionated heparin, CKD, chronic kidney disease, CRE, creatinine, RA, rheumatoid arthritis

**Supplement Table 3. Quality assessment based on AHRQ**

| Study | Item 1 | Item 2 | Item 3 | Item 4 | Item 5 | Item 6 | Item 7 | Item 8 | Item 9 | Item 10 | Item 11 | Total points |
| --- | --- | --- | --- | --- | --- | --- | --- | --- | --- | --- | --- | --- |
| Lippenszky, 2024 | 1 | 1 | 1 | 1 | 1 | 1 | 1 | 0 | 1 | 1 | 1 | 10 |
| Cheng, 2023 | 1 | 1 | 1 | 1 | 1 | 1 | 1 | 1 | 0 | 1 | 1 | 10 |
| Gong, 2023 | 1 | 1 | 1 | 1 | 1 | 1 | 1 | 1 | 1 | 1 | 0 | 10 |
| Heilbroner, 2021 | 1 | 1 | 0 | 0 | 1 | 1 | 1 | 0 | 1 | 1 | 1 | 8 |
| Jiang, 2023 | 1 | 1 | 1 | 1 | 1 | 1 | 1 | 0 | 1 | 1 | 0 | 9 |
| Kim,2021a | 1 | 1 | 1 | 1 | 1 | 1 | 1 | 0 | 0 | 1 | 1 | 9 |
| Lewinson, 2021 | 0 | 0 | 0 | 0 | 0 | 0 | 1 | 1 | 0 | 1 | 1 | 4 |
| Kim,2021b | 1 | 1 | 1 | 1 | 1 | 1 | 1 | 0 | 1 | 1 | 1 | 10 |
| Surendran, 2024 | 1 | 1 | 1 | 1 | 1 | 1 | 1 | 1 | 1 | 1 | 0 | 10 |
| Hu, 2023 | 1 | 1 | 1 | 1 | 1 | 1 | 1 | 0 | 1 | 1 | 0 | 9 |
| Chambers, 2023 | 1 | 1 | 1 | 1 | 1 | 1 | 1 | 1 | 0 | 0 | 1 | 9 |
| Chang, 2022 | 1 | 1 | 1 | 1 | 1 | 1 | 1 | 1 | 1 | 1 | 1 | 11 |
| Huang, 2022 | 1 | 1 | 1 | 1 | 1 | 1 | 1 | 0 | 1 | 1 | 0 | 9 |
| On, 2022 | 1 | 1 | 1 | 1 | 1 | 1 | 1 | 0 | 1 | 1 | 1 | 10 |
| Sarrias, 2023 | 1 | 1 | 1 | 1 | 1 | 1 | 1 | 1 | 1 | 1 | 0 | 10 |
| Satheeshkumar, 2021 | 1 | 1 | 1 | 1 | 1 | 0 | 1 | 0 | 1 | 1 | 0 | 8 |
| Venäläinen，2021 | 1 | 1 | 1 | 1 | 1 | 1 | 1 | 1 | 0 | 1 | 0 | 9 |
| Zhang,2023 | 1 | 1 | 1 | 1 | 1 | 1 | 1 | 0 | 1 | 1 | 0 | 9 |
| Zhou,2021 | 1 | 1 | 1 | 1 | 1 | 1 | 1 | 1 | 1 | 1 | 0 | 10 |
| Li, 2022 | 1 | 1 | 1 | 1 | 1 | 1 | 1 | 1 | 1 | 1 | 0 | 10 |
| Matsumoto, 2024 | 1 | 1 | 1 | 1 | 1 | 1 | 1 | 0 | 0 | 1 | 0 | 8 |
| Huang,2023 | 1 | 1 | 1 | 1 | 1 | 1 | 1 | 0 | 1 | 1 | 0 | 9 |
| Nguyen,2023 | 1 | 1 | 1 | 1 | 1 | 1 | 0 | 0 | 0 | 1 | 0 | 7 |
| Yagi,2024 | 1 | 1 | 1 | 1 | 1 | 1 | 1 | 0 | 1 | 1 | 1 | 10 |
| Chiu, 2022 | 1 | 1 | 1 | 1 | 1 | 1 | 1 | 1 | 1 | 1 | 0 | 10 |
| Okawa, 2022 | 1 | 1 | 1 | 1 | 1 | 1 | 1 | 0 | 0 | 1 | 0 | 8 |
| Imai, 2019 | 1 | 1 | 1 | 1 | 1 | 1 | 1 | 0 | 1 | 1 | 0 | 9 |
| Mu,2022 | 1 | 1 | 1 | 1 | 1 | 1 | 1 | 0 | 1 | 1 | 0 | 9 |
| Imai,2020 | 1 | 1 | 1 | 1 | 1 | 1 | 1 | 0 | 0 | 1 | 0 | 8 |
| Zhao,2024 | 1 | 1 | 1 | 1 | 1 | 1 | 1 | 0 | 0 | 1 | 0 | 8 |
| Patel,2024 | 1 | 1 | 1 | 1 | 1 | 1 | 1 | 0 | 1 | 1 | 1 | 10 |
| Asai, 2023 | 1 | 1 | 1 | 1 | 1 | 1 | 1 | 0 | 1 | 1 | 0 | 9 |
| Ma,2023a | 1 | 1 | 1 | 1 | 1 | 1 | 0 | 0 | 0 | 1 | 0 | 7 |
| Lai,2020 | 1 | 1 | 1 | 1 | 1 | 1 | 1 | 0 | 0 | 1 | 0 | 8 |
| Xiao,2024 | 1 | 1 | 1 | 1 | 1 | 1 | 1 | 0 | 0 | 1 | 0 | 8 |
| Liao,2023 | 1 | 1 | 1 | 1 | 1 | 1 | 1 | 1 | 1 | 1 | 0 | 10 |
| Jeong, 2023 | 1 | 1 | 1 | 1 | 1 | 1 | 1 | 1 | 1 | 1 | 0 | 10 |
| Lee,2022 | 1 | 1 | 1 | 1 | 1 | 1 | 1 | 0 | 1 | 1 | 1 | 10 |
| Liu, 201 | 1 | 1 | 1 | 1 | 1 | 1 | 1 | 1 | 1 | 1 | 0 | 10 |
| Dong,2020 | 1 | 1 | 1 | 1 | 1 | 1 | 0 | 1 | 0 | 1 | 0 | 8 |
| Xu, 2022 | 1 | 1 | 1 | 1 | 1 | 1 | 1 | 0 | 1 | 1 | 0 | 9 |
| Mora,2023 | 1 | 1 | 1 | 1 | 1 | 1 | 1 | 0 | 1 | 1 | 0 | 9 |
| Chen, 2023 | 1 | 1 | 1 | 1 | 1 | 1 | 1 | 0 | 1 | 1 | 1 | 10 |
| Herrin,2021 | 1 | 1 | 1 | 1 | 1 | 1 | 1 | 1 | 1 | 1 | 1 | 11 |
| Choi,2024 | 1 | 1 | 1 | 1 | 1 | 1 | 1 | 0 | 1 | 1 | 0 | 9 |
| Zhou,2023 | 1 | 1 | 1 | 1 | 1 | 1 | 1 | 0 | 0 | 1 | 0 | 8 |
| Sun, 2020 | 1 | 1 | 1 | 1 | 1 | 1 | 1 | 1 | 1 | 1 | 0 | 10 |
| Yan, 2023 | 1 | 1 | 1 | 1 | 1 | 1 | 1 | 1 | 1 | 1 | 1 | 11 |
| Ma, 2023b | 1 | 1 | 1 | 1 | 1 | 1 | 1 | 0 | 1 | 1 | 0 | 9 |
| Heo,2023 | 1 | 1 | 1 | 1 | 1 | 1 | 1 | 0 | 1 | 1 | 1 | 10 |
| Güven,2023 | 1 | 1 | 1 | 1 | 1 | 1 | 1 | 1 | 1 | 1 | 1 | 11 |
| Noda,2023 | 1 | 1 | 1 | 1 | 1 | 1 | 1 | 1 | 1 | 1 | 0 | 10 |
| Mao, 2023 | 1 | 1 | 1 | 1 | 1 | 1 | 1 | 1 | 1 | 1 | 1 | 11 |
| Maray, 2023 | 1 | 1 | 1 | 1 | 1 | 1 | 1 | 0 | 1 | 1 | 1 | 10 |
| Yoo,2020 | 1 | 1 | 1 | 1 | 1 | 1 | 1 | 0 | 0 | 1 | 0 | 8 |
| Lu, 2023 | 1 | 1 | 1 | 1 | 1 | 1 | 1 | 1 | 1 | 1 | 0 | 10 |
| Simon, 2021 | 1 | 1 | 1 | 1 | 1 | 1 | 1 | 1 | 1 | 1 | 0 | 10 |
| Wu, 2022 | 1 | 1 | 1 | 1 | 1 | 1 | 1 | 1 | 1 | 1 | 0 | 10 |
| Goyal,2023 | 1 | 1 | 1 | 1 | 1 | 1 | 1 | 1 | 1 | 1 | 0 | 10 |
| Average score | 0.983 | 0.983 | 0.966 | 0.966 | 0.983 | 0.966 | 0.949 | 0.424 | 0.729 | 0.983 | 0.322 | 9.254 |

Item1: Define the source of information (survey, record review). Item 2: List inclusion and exclusion criteria for exposed and unexposed subjects (cases and controls) or refer to previous publications. Item 3. Indicate time period used for identifying patients. Item 4. Indicate whether or not subjects were consecutive if not population-based. Item 5. Indicate if evaluators of subjective components of study were masked to other aspects of the participants. Item 6. Describe any assessments undertaken for quality assurance purposes (e.g.,test/retest of primary outcome measurements). Item 7. Explain any patient exclusions from analysis. Item 8. Describe how confounding was assessed and/or controlled. Item 9. If applicable, explain how missing data were handled in the analysis. Item 10. Summarize patient response rates and completeness of data collection. Item 11. Clarify what follow-up, if any, was expected and the percentage of patients for which incomplete data or follow-up was obtained.

**Supplement Table 4. Quality assessment based on the ChAMAI checklist**

| Study | Problem understanding (10) | Data understanding (6) | Data preparation (8) | Modeling (6) | Validation (12) | Deployment (8) | Total points (50) |
| --- | --- | --- | --- | --- | --- | --- | --- |
| Lippenszky, 2024 | 10 | 6 | 4 | 6 | 6 | 4 | 36 |
| Cheng, 2023 | 10 | 4 | 3 | 6 | 6 | 3 | 32 |
| Gong, 2023 | 10 | 4 | 4 | 6 | 10 | 4 | 38 |
| Heilbroner, 2021 | 10 | 6 | 2 | 6 | 4 | 3 | 31 |
| Jiang, 2023 | 10 | 4 | 2 | 6 | 5 | 5 | 32 |
| Kim,2021a | 10 | 4 | 0 | 6 | 6 | 3 | 29 |
| Lewinson, 2021 | 8 | 0 | 4 | 6 | 2 | 2 | 22 |
| Kim,2021b | 10 | 4 | 2 | 6 | 8 | 4 | 34 |
| Surendran, 2024 | 10 | 6 | 4 | 6 | 8 | 3 | 37 |
| Hu, 2023 | 10 | 4 | 4 | 6 | 3 | 3 | 30 |
| Chambers, 2023 | 10 | 4 | 2 | 6 | 8 | 2 | 32 |
| Chang, 2022 | 10 | 6 | 4 | 6 | 3 | 3 | 32 |
| Huang, 2022 | 10 | 4 | 2 | 6 | 6 | 3 | 31 |
| On, 2022 | 10 | 6 | 4 | 6 | 5 | 4 | 35 |
| Sarrias, 2023 | 10 | 6 | 8 | 6 | 6 | 4 | 40 |
| Satheeshkumar, 2021 | 10 | 4 | 6 | 6 | 8 | 2 | 36 |
| Venäläinen，2021 | 10 | 6 | 0 | 6 | 3 | 2 | 27 |
| Zhang,2023 | 10 | 4 | 6 | 6 | 8 | 4 | 38 |
| Zhou,2021 | 10 | 5 | 4 | 6 | 6 | 3 | 34 |
| Li, 2022 | 10 | 6 | 4 | 6 | 5 | 3 | 34 |
| Matsumoto, 2024 | 10 | 4 | 0 | 6 | 6 | 3 | 29 |
| Huang,2023 | 10 | 6 | 1 | 6 | 2 | 3 | 28 |
| Nguyen,2023 | 10 | 5 | 0 | 6 | 4 | 2 | 27 |
| Yagi,2024 | 10 | 3 | 2 | 6 | 2 | 4 | 27 |
| Chiu, 2022 | 10 | 6 | 6 | 6 | 6 | 3 | 37 |
| Okawa, 2022 | 10 | 5 | 0 | 6 | 2 | 4 | 27 |
| Imai, 2019 | 10 | 6 | 2 | 6 | 4 | 3 | 31 |
| Mu,2022 | 10 | 5 | 4 | 6 | 6 | 4 | 35 |
| Imai,2020 | 10 | 3 | 0 | 6 | 4 | 3 | 26 |
| Zhao,2024 | 10 | 3 | 2 | 6 | 2 | 4 | 27 |
| Patel,2024 | 10 | 5 | 2 | 6 | 2 | 4 | 29 |
| Asai, 2023 | 10 | 3 | 0 | 6 | 4 | 2 | 25 |
| Ma,2023a | 10 | 3 | 0 | 6 | 5 | 2 | 26 |
| Lai,2020 | 10 | 5 | 0 | 6 | 4 | 2 | 27 |
| Xiao,2024 | 10 | 5 | 0 | 6 | 4 | 3 | 28 |
| Liao,2023 | 10 | 5 | 6 | 6 | 4 | 3 | 34 |
| Jeong, 2023 | 10 | 6 | 4 | 6 | 4 | 3 | 33 |
| Lee,2022 | 10 | 6 | 2 | 6 | 6 | 3 | 33 |
| Liu, 201 | 10 | 5 | 6 | 6 | 4 | 3 | 34 |
| Dong,2020 | 9 | 4 | 4 | 6 | 2 | 3 | 28 |
| Xu, 2022 | 10 | 6 | 2 | 6 | 2 | 3 | 29 |
| Mora,2023 | 10 | 5 | 2 | 6 | 8 | 2 | 33 |
| Chen, 2023 | 10 | 6 | 2 | 6 | 6 | 3 | 33 |
| Herrin,2021 | 10 | 6 | 4 | 6 | 4 | 3 | 33 |
| Choi,2024 | 10 | 4 | 2 | 6 | 8 | 3 | 33 |
| Zhou,2023 | 10 | 6 | 0 | 6 | 2 | 3 | 27 |
| Sun, 2020 | 10 | 4 | 6 | 6 | 4 | 3 | 33 |
| Yan, 2023 | 10 | 6 | 4 | 6 | 7 | 4 | 37 |
| Ma, 2023b | 10 | 6 | 4 | 6 | 4 | 3 | 33 |
| Heo,2023 | 10 | 6 | 2 | 6 | 2 | 4 | 30 |
| Güven,2023 | 10 | 6 | 4 | 6 | 4 | 3 | 33 |
| Noda,2023 | 10 | 6 | 4 | 6 | 6 | 3 | 35 |
| Mao, 2023 | 10 | 6 | 6 | 6 | 6 | 3 | 37 |
| Maray, 2023 | 10 | 6 | 2 | 6 | 2 | 2 | 28 |
| Yoo,2020 | 10 | 4 | 2 | 6 | 4 | 2 | 28 |
| Lu, 2023 | 10 | 6 | 8 | 6 | 6 | 3 | 39 |
| Simon, 2021 | 10 | 6 | 2 | 6 | 4 | 2 | 30 |
| Wu, 2022 | 10 | 6 | 6 | 6 | 4 | 3 | 35 |
| Goyal,2023 | 10 | 6 | 4 | 6 | 6 | 3 | 35 |


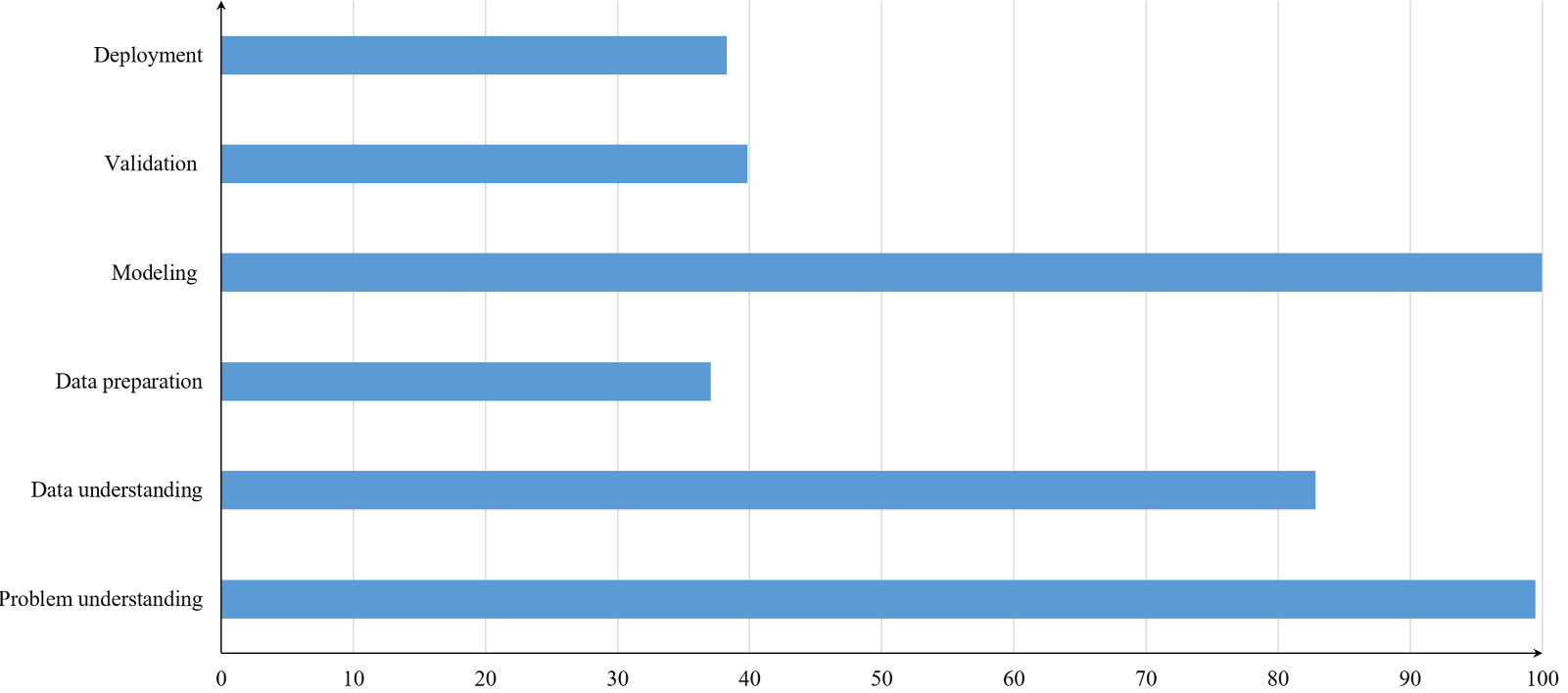
 **Supplement Figure 1 The scoring rate of ChAMAI checklist**

**Supplementary Table 5 Performance of ML in included studies**

| Study ID | ADE | ML Model | Accuracy | Sensitivity | Specificity | Precision | F1-score | AUC | AUPRC |
| --- | --- | --- | --- | --- | --- | --- | --- | --- | --- |
| Lippenszky, 202 | Pneumonitis | RF | N | 0.71 | 0.7 | N | N | 0.74 | N |
|  | Hepatic dysfunction | RF | N | 0.45 | 0.45 | N | N | 0.73 | N |
|  | Colitis | RF | N | 0.75 | 0.61 | N | N | 0.75 | N |
| Cheng, 2023 | Pneumonitis | CNN+LR | 0.93 | 0.75 | 1 | N | N | 0.9 | N |
| Gong, 2023 | Pneumonitis | EN | N | N | N | 0.68 | N | 0.81 | N |
| Heilbroner, 2021 | Cardiac event | XGBoost | N | N | N | N | N | 0.65 | N |
| Jiang, 2023 | Hematological toxicity | DT | 0.59 | 0.21 | 0.83 | N | N | 0.52 | N |
|  |  | RF | 0.68 | 0.36 | 0.87 | N | N | 0.61 | N |
|  |  | GBDT | 0.57 | 0.21 | 0.78 | N | N | 0.5 | N |
|  |  | XGBoost | 0.73 | 0.43 | 0.91 | N | N | 0.67 | N |
|  |  | LightGBM | 0.57 | 0.36 | 0.7 | N | N | 0.53 | N |
|  |  | LR | 0.7 | 0.47 | 0.86 | N | N | 0.66 | N |
| Kim,2021a | Thyroid-Related ADE | LR | N | N | N | N | N | 0.71 | 0.47 |
|  |  | EN | N | N | N | N | N | 0.71 | 0.47 |
|  |  | RF | N | N | N | N | N | 0.77 | 0.51 |
|  |  | SVM (Linear) | N | N | N | N | N | 0.57 | 0.36 |
|  |  | SVM (Radial) | N | N | N | N | N | 0.69 | 0.45 |
| Lewinson, 2021 | Cutaneous ADE | RF | 0.78 | 0.85 | 0.68 | N | N | 0.76 | N |
| Kim,2021b | Hepatic dysfunction | MLR | N | 0.86 | 0.27 | N | N | N | N |
|  |  | EN | N | 0.91 | 0.22 | N | N | N | N |
|  |  | RF | N | 0.75 | 0.38 | N | N | N | N |
|  |  | SVM (Linear) | N | 0.9 | 0.14 | N | N | N | N |
|  |  | SVM (Radial) | N | 0.63 | 0.33 | N | N | N | N |
| Surendran, 2024 | Hepatic dysfunction | RF (select feature) | N | 0.86 | N | 0.81 | N | 0.63 | N |
|  |  | RF (full-feature) | N | 0.87 | N | 0.89 | N | 0.66 | N |
| Hu, 2023 | Hepatic dysfunction | LightGBM | 0.6 | 0.25 | 0.79 | 0.4 | 0.31 | 0.87 | N |
|  |  | GBDT | 0.59 | 0.41 | 0.69 | 0.51 | 0.42 | 0.53 | N |
|  |  | Adaboost | 0.64 | 0.34 | 0.82 | 0.51 | 0.41 | 0.69 | N |
|  |  | Catboost | 0.6 | 0.32 | 0.76 | 0.43 | 0.37 | 0.91 | N |
|  |  | XGboost | 0.6 | 0.34 | 0.75 | 0.43 | 0.38 | 0.94 | N |
|  |  | RF | 0.64 | 0.32 | 0.82 | 0.5 | 0.39 | 0.97 | N |
|  |  | TPOT | 0.61 | 0.32 | 0.77 | 0.44 | 0.37 | 0.78 | N |
|  |  | ANN | 0.62 | 0.07 | 0.07 | 0.36 | 0.12 | 0.65 | N |
| Chambers, 2023 | Renal dysfunction | MLP | N | 0.6 | 0.98 | N | 0.59 | 0.76 | N |
|  | Hepatic dysfunction | MLP | N | 0.54 | 0.9 | N | 0.24 | 0.72 | N |
| Chang, 2022 | Cardiac event | RF | 0.87 | 0 | 0.98 | 0.44 | 0.47 | 0.49 | N |
|  |  | LR | 0.55 | 0.57 | 0.54 | 0.523 | 0.45 | 0.56 | N |
|  |  | SVM | 0.59 | 0.57 | 0.6 | 0.53 | 0.48 | 0.58 | N |
|  |  | KNN | 0.55 | 0.43 | 0.56 | 0.5 | 0.43 | 0.49 | N |
|  |  | LightGBM | 0.89 | 0.14 | 0.98 | 0.7 | 0.58 | 0.56 | N |
|  |  | MLP | 0.56 | 0.86 | 0.53 | 0.57 | 0.49 | 0.66 | N |
| Huang, 2022 | Renal dysfunction | ANN (integrated) | 0.92 | 0.71 | N | 0.95 | 0.81 | 0.9 | N |
|  |  | LR (integrated) | 0.9 | 0.61 | N | 0.96 | 0.72 | 0.89 | N |
|  |  | RF (integrated) | 0.85 | 0.39 | N | 0.95 | 0.54 | 0.87 | N |
|  |  | SVM (integrated) | 0.86 | 0.39 | N | 1 | 0.55 | 0.86 | N |
| On, 2022 | Nausea-vomiting | Logistic regression | 0.83 | N | N | N | N | 0.75 | N |
|  |  | DT | 0.83 | N | N | N | N | 0.81 | N |
|  |  | NN | 0.83 | N | N | N | N | 0.76 | N |
|  | Fatigue-anorexia | Logistic regression | 0.62 | N | N | N | N | 0.59 | N |
|  |  | DT | 0.69 | N | N | N | N | 0.72 | N |
|  |  | NN | 0.66 | N | N | N | N | 0.61 | N |
|  | Diarrhea | Logistic regression | 0.67 | N | N | N | N | 0.71 | N |
|  |  | DT | 0.61 | N | N | N | N | 0.55 | N |
|  |  | NN | 0.65 | N | N | N | N | 0.63 | N |
|  | Peripheral neuropathy | Logistic regression | 0.69 | N | N | N | N | 0.64 | N |
|  |  | DT | 0.66 | N | N | N | N | 0.67 | N |
|  |  | NN | 0.62 | N | N | N | N | 0.57 | N |
|  | Hypersensitivity | Logistic regression | 0.79 | N | N | N | N | 0.76 | N |
|  |  | DT | 0.77 | N | N | N | N | 0.71 | N |
|  |  | NN | 0.66 | N | N | N | N | 0.62 | N |
|  | Stomatitis | Logistic regression | 0.72 | N | N | N | N | 0.65 | N |
|  |  | DT | 0.67 | N | N | N | N | 0.83 | N |
|  |  | NN | 0.68 | N | N | N | N | 0.61 | N |
|  | Hand-foot syndrome | Logistic regression | 0.71 | N | N | N | N | 0.74 | N |
|  |  | DT | 0.67 | N | N | N | N | 0.64 | N |
|  |  | NN | 0.63 | N | N | N | N | 0.6 | N |
|  | Constipation | Logistic regression | 0.72 | N | N | N | N | 0.84 | N |
|  |  | DT | 0.78 | N | N | N | N | 0.88 | N |
|  |  | NN | 0.7 | N | N | N | N | 0.88 | N |
| Sarrias, 2023 | Hematological toxicity | BN | 0.8 | 0.71 | 0.83 | N | N | N | N |
| Satheeshkumar, 2021 | Ulcerative mucositis | lasso | N | N | N | N | N | 0.75 | N |
|  |  | GBM | N | N | N | N | N | 0.79 | N |
| Venäläinen，2021 | Neutropenic infection | lasso | N | N | N | N | N | 0.84 | N |
| Zhang,2023 | Nausea-vomiting | RF | 0.82 | 0.74 | 0.83 | N | 0.71 | 0.84 | N |
|  |  | SVM | 0.79 | 0.69 | 0.81 | N | 0.68 | 0.81 | N |
|  |  | Catboost | 0.81 | 0.84 | 0.8 | N | 0.72 | 0.84 | N |
|  |  | NN | 0.81 | 0.82 | 0.8 | N | 0.71 | 0.84 | N |
|  |  | DT | 0.87 | 0.71 | 0.9 | N | 0.77 | 0.81 | N |
|  |  | DF | 0.85 | 0.78 | 0.86 | N | 0.76 | 0.85 | N |
| Zhou,2021 | Cognitive Impairment | RF | N | N | N | N | N | 0.73 | N |
|  |  | LR | N | N | N | N | N | 0.8 | N |
|  |  | SVM | N | N | N | N | N | 0.77 | N |
| Li, 2022 | Cardiac event | XGBoost | N | N | N | 0.62 | 0.41 | 0.82 | N |
|  |  | LR | N | N | N | 0.61 | 0.4 | 0.81 | N |
|  |  | RF | N | N | N | 0.61 | 0.39 | 0.8 | N |
| Matsumoto, 2024 | Thrombocytopenia | EM combine with GBDT | 0.82 | 0.5 | N | 0.68 | 0.58 | 0.76 | N |
| Huang,2023 | Hepatic dysfunction | ANN | 0.97 | 0.28 | 1 | N | N | 0.92 | N |
|  |  | LR | 0.96 | 0.17 | 0.99 | N | N | 0.88 | N |
| Nguyen,2023 | Cardiac event | LR | N | 0.82 | N | 0.09 | 0.17 | 0.65 | N |
|  |  | ANN | N | 0.83 | N | 0.14 | 0.2 | 0.9 | N |
| Chiu, 2022 | Renal dysfunction | CatBoost | N | 0.51 | 0.86 | N | 0.58 | 0.79 | 0.69 |
|  |  | CatBoost -SMOTE | N | 0.64 | 0.76 | N | 0.62 | 0.78 | 0.69 |
|  |  | CatBoost -SVMSMOTE | N | 0.68 | 0.76 | N | 0.65 | 0.79 | 0.68 |
|  |  | CatBoost -Tomek | N | 0.61 | 0.81 | N | 0.63 | 0.78 | 0.68 |
|  |  | CatBoost -SMOTETL | N | 0.6 | 0.82 | N | 0.63 | 0.77 | 0.68 |
|  |  | LightGBM | N | 0.56 | 0.84 | N | 0.61 | 0.78 | 0.68 |
|  |  | LightGBM -SMOTE | N | 0.74 | 0.66 | N | 0.63 | 0.77 | 0.65 |
|  |  | LightGBM -SVMSMOTE | N | 0.72 | 0.69 | N | 0.63 | 0.77 | 0.66 |
|  |  | LightGBM -Tomek | N | 0.58 | 0.81 | N | 0.6 | 0.78 | 0.68 |
|  |  | LightGBM -SMOTETL | N | 0.69 | 0.72 | N | 0.63 | 0.78 | 0.67 |
|  |  | RF | N | 0.51 | 0.83 | N | 0.56 | 0.77 | 0.65 |
|  |  | RF-SMOTE | N | 0.66 | 0.74 | N | 0.63 | 0.78 | 0.66 |
|  |  | RF-SVMSMOTE | N | 0.66 | 0.72 | N | 0.61 | 0.78 | 0.66 |
|  |  | RF-Tomek | N | 0.62 | 0.77 | N | 0.61 | 0.78 | 0.67 |
|  |  | RF-SMOTETL | N | 0.6 | 0.73 | N | 0.62 | 0.78 | 0.6 |
| Yagi,2024 | Cardiac event | AI-EF model | N | N | N | N | N | 0.78 | N |
| Okawa, 2022 | AKI | NN combine with GBDT | 0.65 | 0.69 | N | 0.22 | 0.33 | 0.67 | N |
| Imai, 2019 | Neutropenia | LR | 0.85 | N | N | N | N | N | N |
|  |  | DT | 0.85 | N | N | N | N | N | N |
| Mu,2022 | AKI | XGBoost | 0.91 | N | N | N | N | 0.88 | 0.83 |
| Imai,2020 | Renal dysfunction | ANN | 0.86 | N | N | N | N | 0.83 | N |
| Zhao,2024 | Thrombocytopenia | ANN | 0.96 | N | N | N | N | 0.94 | N |
|  |  | LR | 0.86 | N | N | N | N | 0.98 | N |
| Patel,2024 | Thrombocytopenia | RFC | 0.85 | 0.76 | N | 0.76 | 0.76 | 0.9 | N |
|  | Anemia | RFC | 0.72 | 0.81 | N | 0.73 | 0.76 | 0.78 | N |
| Asai, 2023 | Hepatic dysfunction | DT | 0.82 | N | N | N | N | N | N |
| Ma,2023a | Hepatic dysfunction | AdaBoost | N | N | N | N | N | 0.74 | N |
|  |  | DT | N | N | N | N | N | 0.72 | N |
|  |  | GBDT | N | N | N | N | N | 0.79 | N |
|  |  | LR | N | N | N | N | N | 0.79 | N |
|  |  | NN | N | N | N | N | N | 0.72 | N |
|  |  | RF | N | N | N | N | N | 0.76 | N |
| Lai,2020 | Hepatic dysfunction | ANN-traditional parameters | 0.78 | 0.48 | 0.9 | N | N | 0.77 | N |
|  |  | ANN-genetic parameters | 0.85 | 0.6 | 0.92 | N | N | 0.84 | N |
|  |  | ANN-combined parameters | 0.89 | 0.8 | 0.9 | N | N | 0.9 | N |
|  |  | SVM-traditional parameters | 0.7 | 0.44 | 0.74 | N | N | 0.69 | N |
|  |  | SVM-genetic parameters | 0.71 | 0.44 | 0.76 | N | N | 0.71 | N |
|  |  | SVM-combined parameters | 0.73 | 0.48 | 0.78 | N | N | 0.73 | N |
|  |  | RF-traditional parameters | 0.69 | 0.48 | 0.73 | N | N | 0.69 | N |
|  |  | RF-genetic parameters | 0.7 | 0.48 | 0.75 | N | N | 0.7 | N |
|  |  | RF-combined parameters | 0.71 | 0.48 | 0.76 | N | N | 0.72 | N |
| Xiao,2024 | Hepatic dysfunction | LR | 0.75 | 0.77 | 0.75 | 0.37 | 0.5 | 0.85 | 0.67 |
|  |  | RF | 0.8 | 0.78 | 0.8 | 0.43 | 0.56 | 0.88 | 0.73 |
|  |  | XGBoost | 0.81 | 0.76 | 0.82 | 0.45 | 0.57 | 0.89 | 0.75 |
| Liao,2023 | Hepatic dysfunction | MLP | 0.85 | 0.75 | 0.92 | N | N | 0.91 | N |
|  |  | RF | 0.86 | 0.79 | 0.9 | N | N | 0.92 | N |
|  |  | LightGBM | 0.85 | 0.77 | 0.9 | N | N | 0.91 | N |
|  |  | XGBoost | 0.87 | 0.78 | 0.92 | N | N | 0.92 | N |
|  |  | SVM | 0.72 | 0.72 | 0.71 | N | N | 0.77 | N |
|  |  | LR | 0.78 | 0.78 | 0.77 | N | N | 0.86 | N |
|  | Acute Respiratory Failure | MLP | 0.81 | 0.81 | 0.79 | N | N | 0.87 | N |
|  |  | RF | 0.82 | 0.81 | 0.82 | N | N | 0.88 | N |
|  |  | LightGBM | 0.81 | 0.75 | 0.81 | N | N | 0.86 | N |
|  |  | XGBoost | 0.81 | 0.81 | 0.81 | N | N | 0.86 | N |
|  |  | SVM | 0.73 | 0.72 | 0.73 | N | N | 0.8 | N |
|  |  | LR | 0.81 | 0.78 | 0.81 | N | N | 0.86 | N |
|  | Mortality | MLP | 0.73 | 0.72 | 0.74 | N | N | 0.83 | N |
|  |  | RF | 0.71 | 0.72 | 0.71 | N | N | 0.81 | N |
|  |  | LightGBM | 0.7 | 0.69 | 0.71 | N | N | 0.81 | N |
|  |  | XGBoost | 0.7 | 0.69 | 0.71 | N | N | 0.81 | N |
|  |  | SVM | 0.69 | 0.78 | 0.68 | N | N | 0.81 | N |
|  |  | LR | 0.66 | 0.67 | 0.66 | N | N | 0.74 | N |
| Jeong, 2023 | Gastric Ulcer | LR | N | 0.45 | 0.82 | N | N | 0.64 | N |
|  |  | SVM | N | 0.57 | 0.71 | N | N | 0.64 | N |
|  |  | RF | N | 0.89 | 0.84 | N | N | 0.86 | N |
|  |  | GBM | N | 0.944 | 0.847 | N | N | 0.896 | N |
|  |  | XGBoost | N | 0.923 | 0.863 | N | N | 0.893 | N |
| Lee,2022 | Gastrointestinal complications | Lasso | N | 0.7 | 0.73 | N | N | 0.79 | N |
| Liu, 2018 | Cardiac event | LR | N | 0.69 | N | 0.75 | N | 0.88 | N |
|  |  | SVM | N | 0.75 | N | 0.64 | N | 0.87 | N |
|  |  | DT | N | 0.5 | N | 0.97 | N | 0.86 | N |
|  |  | GBDT | N | 0.95 | N | 0.89 | N | 0.89 | N |
|  |  | XGBoost | N | 0.84 | N | 0.81 | N | 0.92 | N |
| Dong,2020 | ADE | SPARCS-RF | 0.9603 | 0.64 | N | 0.89 | 0.75 | 0.95 | N |
|  |  | SPARCS-DT | 0.95 | 0.72 | N | 0.76 | 0.72 | 0.85 | N |
|  |  | SPARCS-LR | 0.96 | 0.63 | N | 0.97 | 0.63 | 0.81 | N |
|  |  | SPARCS-DNN | 0.97 | 0.67 | N | 0.97 | 0.67 | 0.94 | N |
|  |  | HF-RF | 0.99 | 0.86 | N | 0.95 | 0.9 | 0.95 | N |
|  |  | HF-DT | 0.97 | 0.83 | N | 0.87 | 0.85 | 0.93 | N |
|  |  | HF-LR | 0.96 | 0.55 | N | 0.96 | 0.7 | 0.74 | N |
|  |  | HF- DNN | 0.98 | 0.78 | N | 0.99 | 0.87 | 0.95 | N |
| Xu, 2022 | Hemorrhage | LR | N | N | N | N | N | 0.79 | N |
|  |  | RF | N | N | N | N | N | 0.7 | N |
| Mora,2023 | Hemorrhage | XGBoost | N | 0.91 | 0.91 | 0.9 | 0.9 | 0.91 | N |
| Chen, 2023 | Hemorrhage | XGBoost | 0.77 | 0.8 | N | 0.92 | 0.86 | 0.78 | N |
| Herrin,2021 | Gastrointestinal hemorrhage | XGBoost | N | 0.59 | 0.66 | N | N | 0.66 | N |
|  |  | RSF | N | 0.58 | 0.58 | N | N | 0.6 | N |
|  |  | RegCox | N | 0.7 | 0.54 | N | N | 0.66 | N |
| Choi,2024 | AKI | Lasso | N | N | N | N | N | 0.87 | 0.37 |
|  |  | GBM | N | N | N | N | N | 0.87 | 0.37 |
|  |  | RF | N | N | N | N | N | 0.85 | 0.36 |
|  |  | DT | N | N | N | N | N | 0.82 | 0.31 |
|  |  | Adaboost | N | N | N | N | N | 0.85 | 0.36 |
| Zhou,2023 | AKI | LR | 0.75 | 0.69 | N | 0.14 | 0.23 | 0.75 | N |
|  |  | NB | 0.76 | 0.61 | N | 0.13 | 0.217 | 0.77 | N |
|  |  | RF | 0.95 | 0.03 | N | 0.5 | 0.05 | 0.74 | N |
|  |  | GDBT | 0.94 | 0.14 | N | 0.36 | 0.2 | 0.75 | N |
|  |  | XGBoost | 0.91 | 0.17 | N | 0.16 | 0.16 | 0.72 | N |
| Sun, 2020 | AKI | RF | 0.72 | 0.65 | 0.73 | 0.31 | 0.42 | N | N |
|  |  | SVM | 0.67 | 0.72 | 0.66 | 0.28 | 0.4 | N | N |
|  |  | LR | 0.22 | 0.632 | 0.15 | 0.12 | 0.2 | N | N |
| Yan, 2023 | AKI | LR | N | N | N | N | N | 0.74 | 0.29 |
|  |  | DNN | N | N | N | N | N | 0.94 | 0.62 |
| Ma, 2023b | Renal dysfunction | DT | N | N | N | N | N | 0.7 | N |
|  |  | KNN | N | N | N | N | N | 0.61 | N |
|  |  | LR | N | N | N | N | N | 0.69 | N |
|  |  | SVM | N | 0.66 | 0.8 | N | N | 0.78 | N |
|  |  | NB | N | N | N | N | N | 0.61 | N |
|  |  | RR | N | N | N | N | N | 0.73 | N |
|  |  | RF | N | N | N | N | N | 0.66 | N |
|  |  | XGBoost | N | N | N | N | N | 0.69 | N |
| Heo,2023 | Hepatic dysfunction | IMV-LSTM module | 0.85 | N | N | 0.79 | 0.71 | 0.87 | 0.76 |
| Güven,2023 | Renal dysfunction | KNN | N | 0.96 | 0.97 | 0.92 | 0.94 | 0.99 | N |
|  |  | SVM | N | 0.84 | 0.95 | 0.87 | 0.85 | 0.96 | N |
|  |  | RF | N | 0.94 | 0.99 | 0.99 | 0.96 | 0.99 | N |
|  |  | NN | N | 0.94 | 0.97 | 0.93 | 0.94 | 0.98 | N |
|  |  | NB | N | 0.7 | 0.93 | 0.78 | 0.74 | 0.89 | N |
|  |  | LR | N | 0.89 | 0.83 | 0.68 | 0.77 | 0.89 | N |
|  |  | XGBoost | N | 0.97 | 0.98 | 0.95 | 0.96 | 0.99 | N |
| Noda,2023 | Renal dysfunction | LR-Original+Feature Selection | 0.69 | 0.57 | 0.71 | N | N | 0.7 | N |
|  |  | LR-backward feature selection+SMOTE | 0.77 | 0.65 | 0.79 | N | N | 0.75 | N |
|  |  | Ridge regularised LR-SMOTE+FeatureSelection | 0.74 | 0.59 | 0.77 | N | N | 0.74 | N |
|  |  | Ridge regularised LR-backward feature selection +Original | 0.7 | 0.64 | 0.72 | N | N | 0.76 | N |
|  |  | Lasso+Original | 0.7 | 0.49 | 0.73 | N | N | 0.63 | N |
|  |  | RF+Original+ FeatureSelection | 0.8 | 0.57 | 0.83 | N | N | 0.78 | N |
|  |  | SVM(Linear)-Original+FeatureSelection | 0.75 | 0.7 | 0.75 | N | N | 0.76 | N |
|  |  | SVM(Polynomial)-Original+FeatureSelection | 0.75 | 0.68 | 0.76 | N | N | 0.76 | N |
|  |  | Adaboost-Original+ FeatureSelection | 0.79 | 0.66 | 0.81 | N | N | 0.77 | N |
|  |  | Logitboost+SMOTE | 0.67 | 0.64 | 0.68 | N | N | 0.68 | N |
|  |  | Random under sampling boost-Original+ FeatureSelection | 0.7 | 0.7 | 0.7 | N | N | 0.75 | N |
|  |  | RF-Original+ FeatureSelection | 0.792 | 0.57 | 0.83 | N | N | 0.78 | N |
| Mao, 2023 | Peripheral neuropathy | XGBoost | 0.82 | 0.75 | 0.86 | 0.75 | 0.75 | 0.89 | N |
|  |  | ET | 0.8 | 1 | 0.67 | 0.67 | 0.8 | 0.83 | N |
|  |  | GBDT | 0.8 | 1 | 0.67 | 0.67 | 0.8 | 0.87 | N |
|  |  | LR | 0.8 | 0.67 | 0.89 | 0.8 | 0.73 | 0.74 | N |
|  |  | RF | 0.73 | 0.83 | 0.67 | 0.62 | 0.71 | 0.91 | N |
| Maray, 2023 | ADE | LR | 0.79 | 1 | 0.69 | N | N | 0.91 | N |
|  |  | CART | 0.81 | 0.8 | 0.7 | N | N | 0.79 | N |
| Yoo,2020 | Sleep side effects | SVM | 0.67 | 0.4 | 0.86 | N | N | 0.63 | N |
|  |  | J48 | 0.86 | 0.87 | 0.86 | N | N | 0.92 | N |
|  |  | LR | 0.69 | 0.47 | 0.86 | N | N | 0.7 | N |
| Lu, 2023 | Thyroid Dysfunction | XGBoost-ENN | 0.92 | 0.7 | N | 0.62 | 0.66 | 0.94 | 0.74 |
|  |  | AdaBoost-Raw | 0.92 | 0.52 | N | 0.7 | 0.59 | 0.92 | 0.64 |
|  |  | KNN-Raw | 0.9 | 0.15 | N | 0.76 | 0.25 | 0.83 | 0.5 |
|  |  | LR-ENN | 0.85 | 0.23 | N | 0.3 | 0.26 | 0.811 | 0.3 |
| Simon, 2021 | Long QT syndrome | RF | N | N | N | N | 0.38 | 0.69 | N |
|  |  | LR | N | N | N | N | 0.36 | 0.65 | N |
|  |  | NB | N | N | N | N | 0.38 | 0.67 | N |
|  |  | DNN | N | N | N | N | 0.4 | 0.71 | N |
| Wu, 2022 | ADE | AdaBoost | 0.76 | 0.54 | N | 0.43 | 0.46 | 0.7 | N |
|  |  | Bagging | 0.78 | 0.49 | N | 0.46 | 0.45 | 0.75 | N |
|  |  | Bernoulli NB | 0.77 | 0.54 | N | 0.44 | 0.47 | 0.72 | N |
|  |  | DT | 0.74 | 0.49 | N | 0.39 | 0.42 | 0.67 | N |
|  |  | EM | 0.81 | 0.58 | N | 0.39 | 0.54 | 0.79 | N |
|  |  | ET | 0.7 | 0.39 | N | 0.31 | 0.33 | 0.6 | N |
|  |  | Gaussian NB | 0.72 | 0.54 | N | 0.36 | 0.43 | 0.67 | N |
|  |  | GBM | 0.78 | 0.52 | N | 0.49 | 0.48 | 0.72 | N |
|  |  | KNN | 0.74 | 0.35 | N | 0.39 | 0.32 | 0.65 | N |
|  |  | LDA | 0.77 | 0.56 | N | 0.46 | 0.49 | 0.72 | N |
|  |  | LR | 0.77 | 0.58 | N | 0.46 | 0.5 | 0.73 | N |
|  |  | Multinomial NB | 0.753 | 0.57 | N | 0.45 | 0.47 | 0.73 | N |
|  |  | Passive aggressive | 0.701 | 0.56 | N | 0.36 | 0.42 | 0.69 | N |
|  |  | QDA | 0.774 | 0.44 | N | 0.43 | 0.41 | 0.66 | N |
|  |  | RF | 0.792 | 0.43 | N | 0.53 | 0.44 | 0.74 | N |
|  |  | SGD | 0.762 | 0.51 | N | 0.45 | 0.43 | 0.72 | N |
|  |  | SVM | 0.792 | 0.44 | N | 0.53 | 0.45 | 0.73 | N |
|  |  | XGBoost | 0.79 | 0.51 | N | 0.51 | 0.49 | 0.74 | N |
| Goyal,2023 | Hemorrhage | Cl-LR-With Feature Selection | 0.6 | 0.64 | 0.59 | N | 0.27 | 0.64 | N |
|  |  | Wa-XGBoost-With Feature Selection | 0.65 | 0.65 | 0.66 | N | 0.37 | 0.68 | N |
|  |  | Ci-RF-With Feature Selection | 0.64 | 0.67 | 0.67 | N | 0.26 | 0.7 | N |
|  |  | Es-RF-With Feature Selection | 0.62 | 0.64 | 0.62 | N | 0.18 | 0.66 | N |
|  |  | Fluo-DT-Withot Feature Selection | 0.81 | 0.37 | 0.85 | N | 0.24 | 0.66 | N |
|  |  | Fluv-XGBoost-With Feature Selection | N | N | N | N | N | 0.77 | N |
|  |  | Pa-RF-With Feature Selection | 0.67 | 0.58 | 0.68 | N | 0.23 | 0.63 | N |
|  |  | Se-RF-Without Feature Selection | 0.62 | 0.67 | 0.62 | N | 0.23 | 0.66 | N |
|  |  | Vo-LR-With Feature Selection | N | N | N | N | N | 0.8 | N |
|  |  | Combined SSRI-XGBoost-Without Feature Selection | 0.7 | 0.59 | 0.71 | N | 0.22 | 0.67 | N |

Type of ADE: NV, nausea-vomiting, FA, fatigue-anorexia, DI, diarrhea, PN, peripheral neuropathy, HS, hypersensitivity, ST, stomatitis, HFS, hand-foot syndrome, CO, constipation, AKI, Acute Kidney Injury, ADE, Adverse drug event.

Drugs that cause ADE: ICI, Immune checkpoint inhibitor, BTK, Bruton's tyrosine kinase MTX, methotrexate, Cl, Clopidogre, Wa, Warfarin, Ci, Citalopram, Es, Escitalopram, Fluo, Fluoxetine, Fluv, Fluvoxamine, Pa, Paroxetine, Se, Sertraline, Vo, Vortioxetine, SSRI, selective serotonin reuptake inhibitor

Machine learning Algorithms: ML Model, Machine learning Model, AUC, area under the curve (AUC) of the receiver operating characteristic curve (ROC), AUPRC, Area Under the Precision-Recall Curve, RF, random forest, CNN, convolutional neural network, LR, Logistic regression, Lasso, Least Absolute Shrinkage and Selection Operator, EN, Elastic Net, XGBoost, eXtreme Gradient Boosting, DT, decision tree, GBDT, gradient boosting decision tree, LightGBM, light gradient boosting machine, AdaBoost, Adaptive Boosting, CatBoost, categorical boosting, TPOT, Tree-based Pipeline Optimization Tool, NN, Neural network, ANN, Artificial Neural Network, SVM, Support vector machine, MLR, Multivariate logistic regression, MLP, Multi-Layer perceptron, KNN, K-Nearest Neighbors, BN, Bayesian Network, NB, Naïve Bayes, DF, Deep forest, EM, ensemble model, AI-EF model, AI model detecting reduced left ventricular ejection fraction from 12-lead electrocardiograms (ECG), RegCox, Cox regression, RSF, random survival forests, ET, extremely random tree, CART, classification and regression trees, GBM, gradient boosting machine, DNN, Deep neural networks, LDA, Latent Dirichlet Allocation, QDA, Quadratic Discriminant Analysis, SGD, Stochastic Gradient Descent, STROBE, Strengthening The Reporting of Observational Studies in Epidemiology, ENN, edited nearest neighbor, B-SMT-ENN, borderline synthetic minority oversampling technique–edited nearest neighbor, SMOTE, synthetic minority oversampling technique, Tomek, Tomek links, SMOTETL, SMOTE and Tomek link, SVM-SMOTE, SVM and SMOTE, IMV-LSTM, interpretability multivariate long short-term memory

**
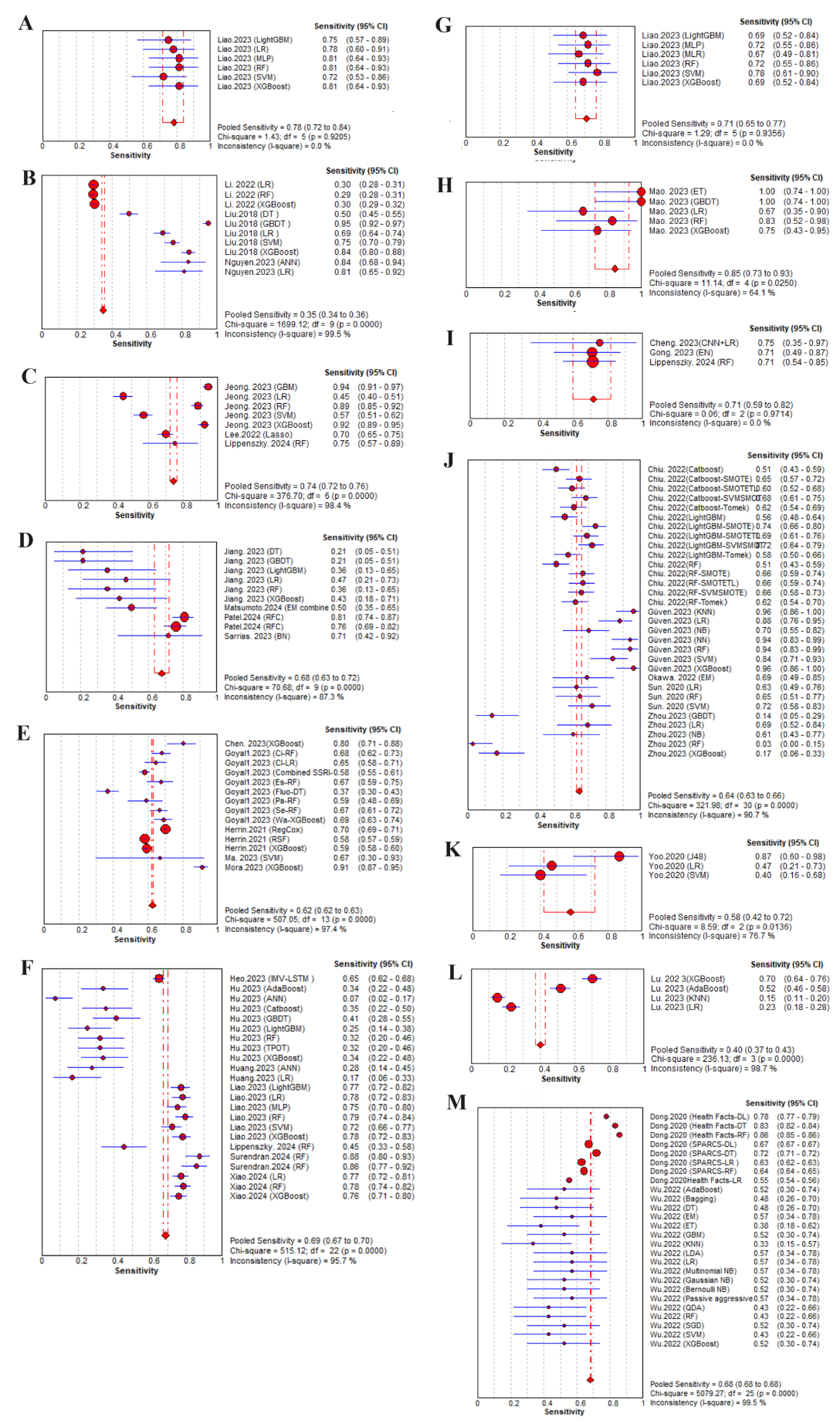
Supplementary Figure 2. Pooled sensitivity of the prediction ADE.** A) Acute Respiratory Failure, B) Cardiac event, C) Gastrointestinal complications, D) Hematological toxicity, E) Hemorrhage, F) Hepatic dysfunction, G) Mortality, H) Peripheral neuropathy, I) Pneumonitis, J) Renal dysfunction, K) Sleep side effects, L) Thyroid Dysfunction, M) ADE

**
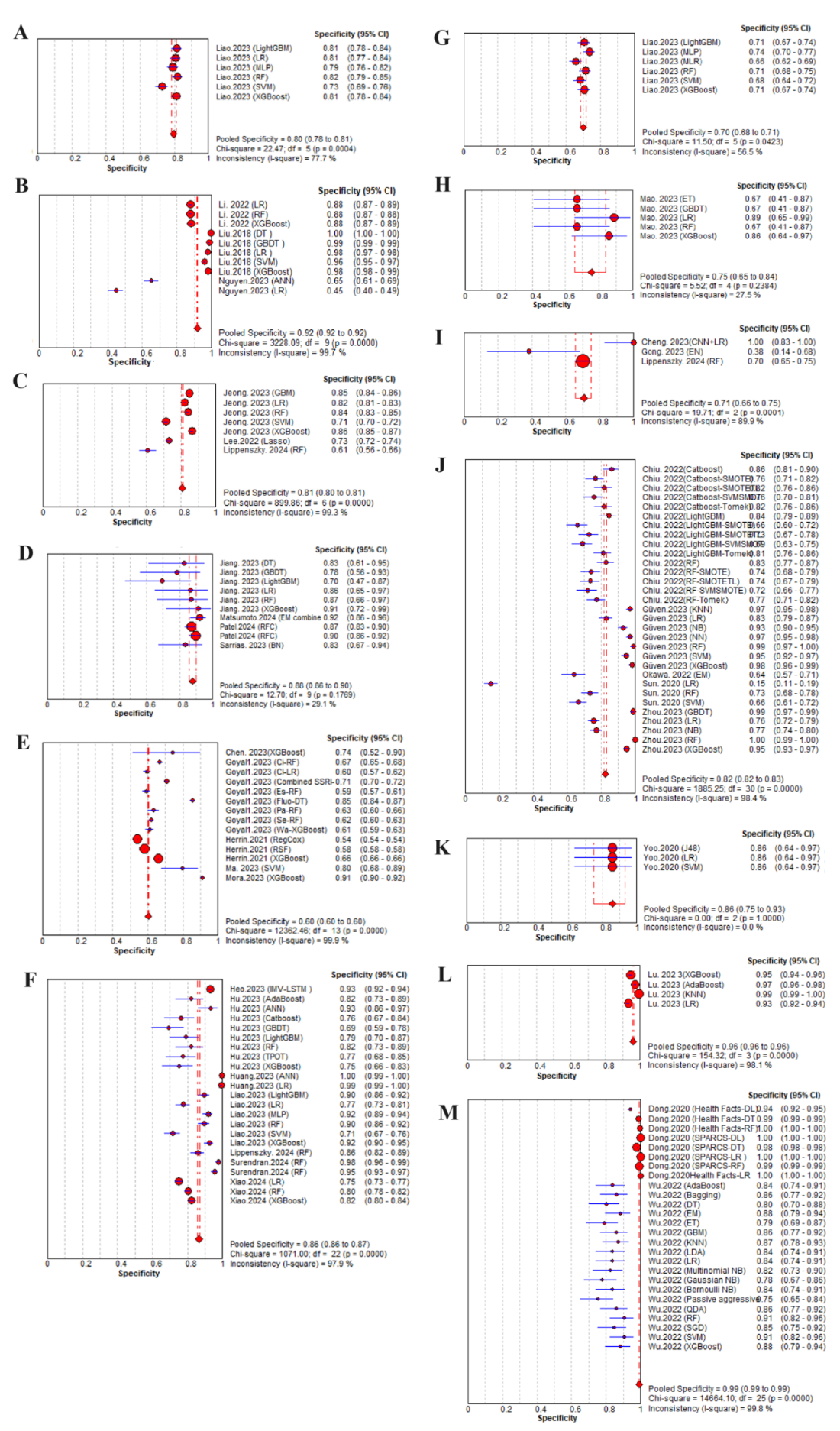
Supplementary Figure 3. Pooled specificity in different ADE.** A) Acute Respiratory Failure, B) Cardiac event, C) Gastrointestinal complications, D) Hematological toxicity, E) Hemorrhage, F) Hepatic dysfunction, G) Mortality, H) Peripheral neuropathy, I) Pneumonitis, J) Renal dysfunction, K) Sleep side effects, L) Thyroid Dysfunction, M) ADE

**
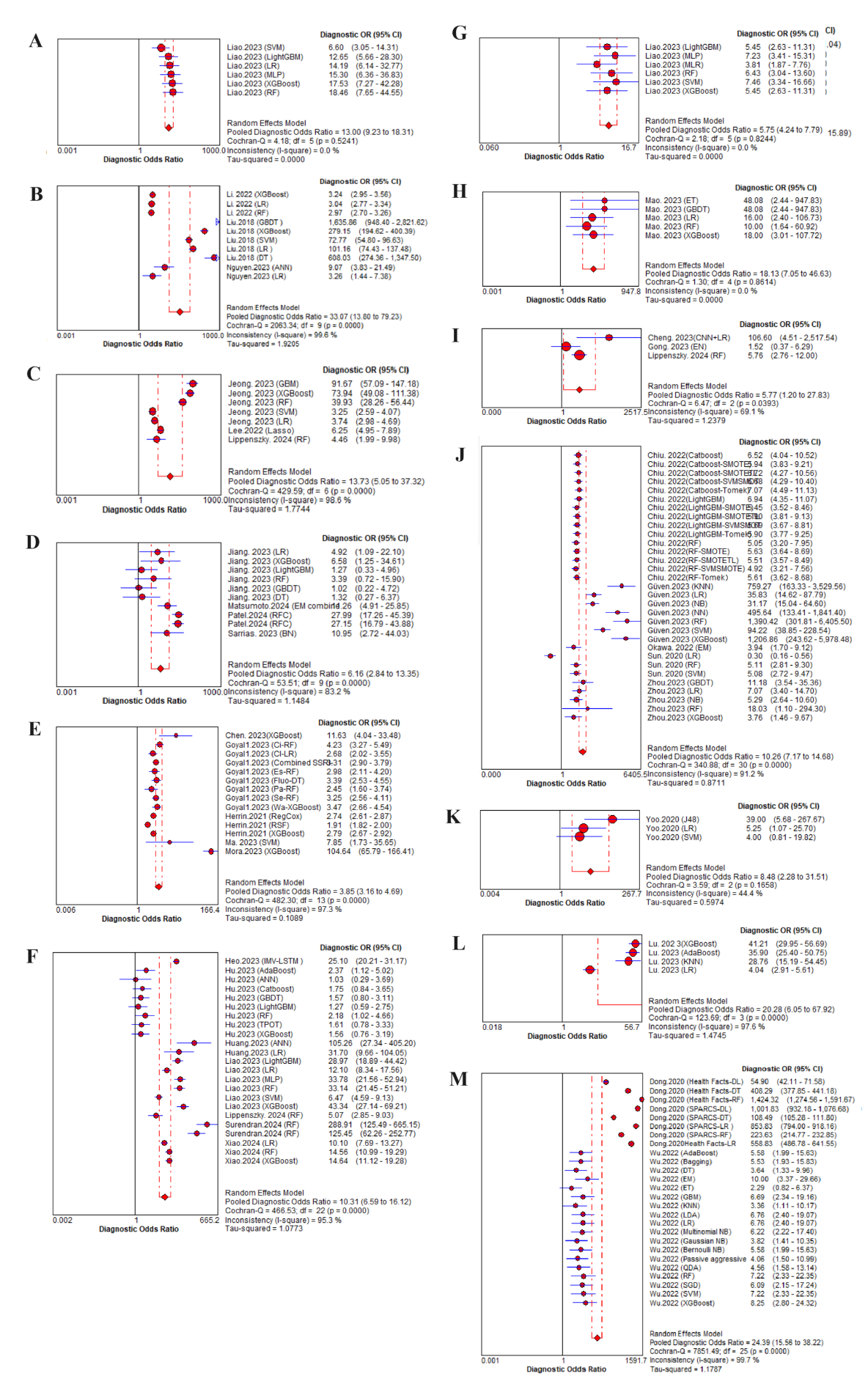
Supplementary Figure 4. Pooled DOR in different ADE.** A) Acute Respiratory Failure, B) Cardiac event, C) Gastrointestinal complications, D) Hematological toxicity, E) Hemorrhage, F) Hepatic dysfunction, G) Mortality, H) Peripheral neuropathy, I) Pneumonitis, J) Renal dysfunction, K) Sleep side effects, L) Thyroid Dysfunction, M) ADE

**
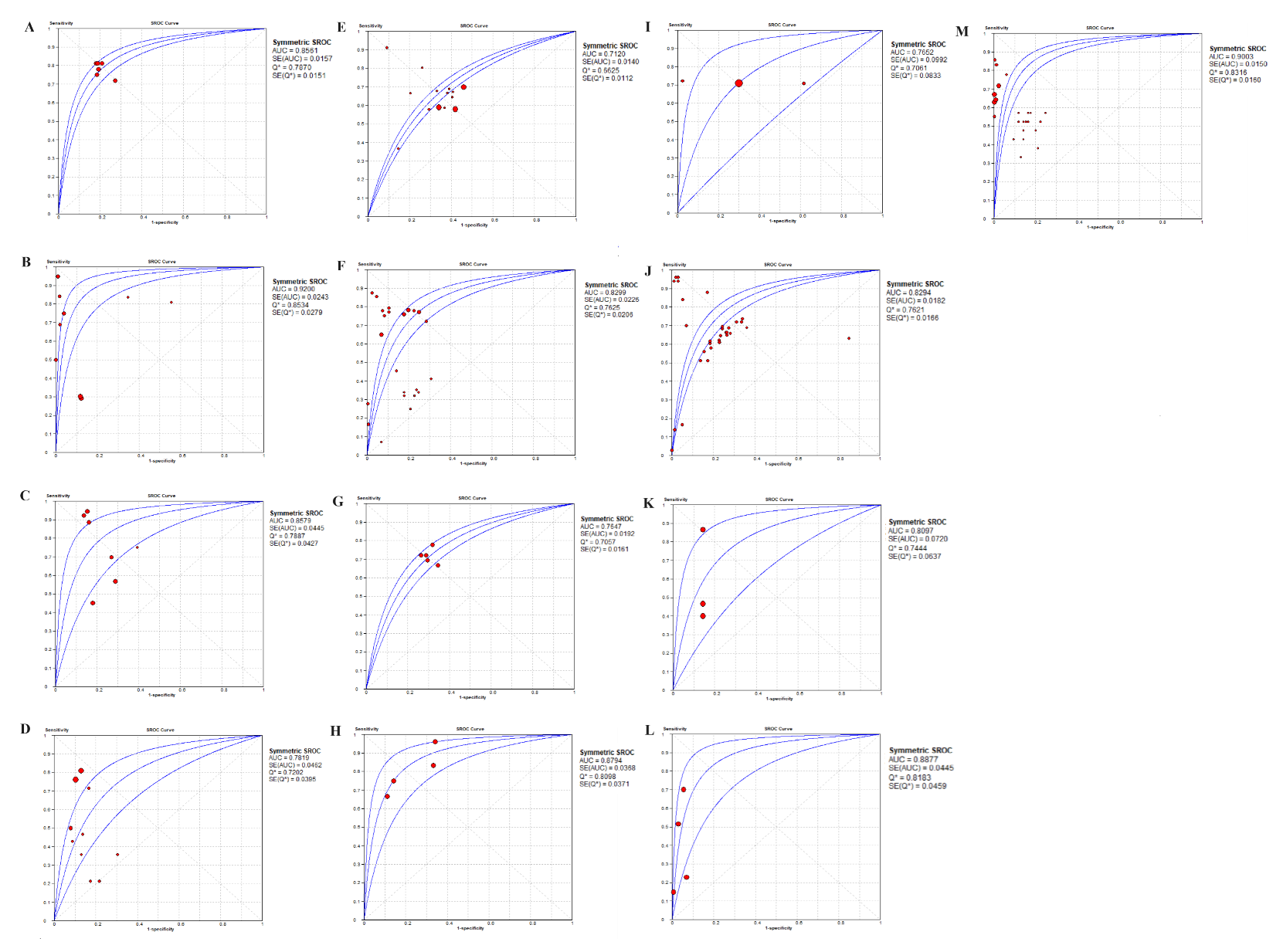
Supplementary Figure 5. Pooled SROC in different ADE.** A) Acute Respiratory Failure, B) Cardiac event, C) Gastrointestinal complications, D) Hematological toxicity, E) Hemorrhage, F) Hepatic dysfunction, G) Mortality, H) Peripheral neuropathy, I) Pneumonitis, J) Renal dysfunction, K) Sleep side effects, L) Thyroid Dysfunction, M) ADE

**Supplementary Table 6. Pooled sensitivity in different ML model.**

| ML model | Sensitivity | 95% CI | *I^2^* |
| --- | --- | --- | --- |
| AdaBoost | 0.49 | 0.44-0.54 | 66.9 |
| ANN | 0.35 | 0.27-0.44 | 96.8 |
| Catboost | 0.6 | 0.56-0.63 | 79.4 |
| DT | 0.71 | 0.71-0.72 | 99.5 |
| EM | 0.64 | 0.49-0.77 | 0.00 |
| ET | 0.61 | 0.42-0.77 | 93.9 |
| GBDT | 0.81 | 0.77-0.84 | 98.1 |
| GBM | 0.92 | 0.88-0.95 | 96.2 |
| KNN | 0.28 | 0.23-0.33 | 98.5 |
| LightGBM | 0.66 | 0.64-0.69 | 88.9 |
| LR | 0.59 | 0.58-0.59 | 99.2 |
| MLP | 0.75 | 0.7-0.79 | 0.00 |
| NB | 0.61 | 0.53-0.69 | 0.00 |
| RF | 0.64 | 0.65-0.66 | 99.4 |
| RFC | 0.79 | 0.74-0.83 | 11.8 |
| SVM | 0.68 | 0.66-0.71 | 81.2 |
| XGBoost | 0.53 | 0.53-0.54 | 99.1 |

AdaBoost, Adaptive Boosting, ANN, Artificial Neural Network, CatBoost, categorical boosting, DT, decision tree, EM, ensemble model, ET, extremely random tree, GBDT, gradient boosting decision tree, GBM, gradient boosting machine, KNN, K-Nearest Neighbors, LightGBM, light gradient boosting machine, LR, Logistic regression, MLP, Multi-Layer perceptron, NB, Naïve Bayes, RF, random forest, RFC, random forest classification SVM, Support vector machine, XGBoost, eXtreme Gradient Boosting
